# Design and fabrication of an aluminium oxide cutting insert with an internal cooling channel

**DOI:** 10.1007/s40436-024-00483-3

**Published:** 2024-03-13

**Authors:** John O’Hara, Feng-Zhou Fang

**Affiliations:** 1 Centre of Micro/Nano Manufacturing Technology (MNMT-Dublin), University College Dublin, Dublin 4, Ireland; 2 State Key Laboratory of Precision Measuring Technology and Instruments, Laboratory of Micro/Nano Manufacturing Technology (MNMT), Tianjin University, Tianjin, 300072 People’s Republic of China

**Keywords:** Additive manufacturing (AM), Aluminium oxide, Cutting insert design, Internal cooling

## Abstract

This paper presents the design and fabrication of an aluminium oxide cutting insert with an internal cooling channel formed through an additive manufacturing method. The formed insert is subjected to a controlled densification process and analysed through a series of characterisation investigations. The purpose of the study is to develop the design concept and analyse the forming and sintering parameters used in the lithographic ceramic manufacturing process. The results validated the feasibility of the geometrical design, providing the required structural conformity with the integrated internal feature using conditional specifications. It is confirmed that the forming parameters would affect the material properties of the green body. Furthermore, the results indicate that the heating rate and temperature variance of the de-binding and thermal treatment regime influences the microstructural growth kinetics and the quality of the densified insert. Using a novel application of liquid gallium as an internal coolant, experimental results showed a decrease in tool wear difference of 36% at $V_{{\text{c}}}$ = 250 m/min, and 31% in tool wear difference at $V_{{\text{c}}}$ = 900 m/min between cooling and non-cooling conditions. When external cooling was applied, the results showed at $V_{{\text{c}}}$ = 250 m/min, the difference between the tool wear rates with the internal coolant relative to the external coolant was 29%. Increasing to $V_{{\text{c}}}$ = 900 m/min, the results revealed a 16% tool wear difference. The results clearly indicate the potential of liquid gallium as a heat transfer agent in internal cooling applications for cutting inserts, and by extension demonstrable reduction in tool wear.

## Introduction

Ceramic fabrication using additive manufacturing (AM) is a developing technology that allows the rapid processing of complex 3D structures designed via a CAD model across diverse fields including micro-mechanics, microfluidics, and micro-optics [1–3]. The process uses a sequential top-down assembly of dimensionally pre-determined layers resulting in a controlled deposition of curable materials in physical space representing the original design, namely, the green body. In ceramic AM, the green body is then subjected to a controlled thermal treatment phase producing a fully densified ceramic component. Although the process allows for the formation of complex shapes that cannot be manufactured through conventional techniques, it does however present challenges in terms of the quality and reproducibility compared to established methods [4, 5]. Nevertheless, the advantages ceramic AM offer, e.g., complex structural features, make it a useful tool to create prototype designs for concept validation.

Widely used in the medical, aerospace and precision manufacturing industries, austenitic stainless steel 316L exhibits excellent physiochemical properties which make it a material of choice for applications requiring high hardness, corrosion resistance and biocompatibility [6, 7]. It is however, as a difficult-to-machine material, and aside from its high hardness, it also has poor thermal conductivity, high tensile strength and produces high abrasion in the cutting tools [8]. This results in a high heat transfer rate from the workpiece back into the cutting tools. Abrasion in particular, both flank and crater wear, occur in the tool with the presence of hard particles within the 316L matrix contributing to the high cutting temperatures. This form of abrasive wear has a signature abrasive profile parallel to the flow chip removal direction [9]. Therefore, a particular type of machining approach is required to address this issue.

Aluminium oxide (Al_2_O_3_), is a widely used oxide ceramic material, characterised by high hardness, chemical inertness, and excellent thermal resistance [10]. Its exceptional physicochemical properties allow for successful applications in the precision machining of hard to cut materials such as 316L [6]. Although ceramics cutting tools can be used in dry machining conditions, the use of cooling methods to enhance the heat transfer in the cutting zone has shown the improvement of the tool wear profile and workpiece surface finish [11, 12].

Previous studies have shown that the use of internal cooling methods to remove heat from the tool edge offer promising results [5, 13–15]. To apply an internal cooling method to a ceramic material, traditional manufacturing methods (such as molding or, casting) are not feasible, whereas AM offers a flexibility not afforded by conventional forming processes.

There has been much interest by researchers in developing new methods to reduce the heat in the cutting tool using internal cooling mechanisms [16, 17]. Closed internal cooling techniques offer an encapsulated system approach to remove the heat during the cutting process [17].

In tandem with the tool insert is the concept of modular design in cutting tools. This method affords a systematic holistic approach as the tool insert cooling unit and toolholder body are now defined and distinct allowing for separation of the parts and ease of replacement [17].

What follows is a review of current developments in internal cooling techniques. Wu et al. [17] built a 3D finite element model (FEM) of the internal cooling system, thereby assessing the thermal performance of the proposed design. The authors used purified liquid water coolant with a 10 mm/s velocity under applied heat conditions of 50 W/mm^2^ over 1 mm^2^ contact area. Results showed the local tool tip temperature without the applied coolant was 155.8 °C and with the coolant active, 67.9 °C was observed.

Yao et al. [18] using purified water as a coolant, designed a closed internal cooling system driven by a mechanical pump in a carbide insert. Numerical modelling simulated the fluidic flow and corresponding temperatures at the tool tip. A thermal imager measured the average temperature distribution across the tool tip, indicating a drop from 433.5 °C to 258.5 °C. This represents a 30% drop in the maximum temperature at the tool tip when compared to dry cutting. However, both methods outlined used liquid water as the cooling agent. The limitations are the thermal conductivity and specific heat capacity of the water.

The geometrical specifications of the internal channel and its proximity to the cutting edge of the tool is an important factor in optimising the design of the internal cooling system. Li et al. [19] designed a topological internal channel that considered the cutting tool dimensions in the design process. In their study, the authors showed that through optimisation of the internal cooling channel, the heat transfer effectiveness could be improved by a margin of 16.32 °C relative to a conventional design, using a flow rate of 1 mm/s through a liquid water coolant. The results emphasise the importance of the geometry and position of the internal channel relative to the heat source when designing the cooling system.

Fang and Obikawa [14], employed a differential pressure flow using high pressure liquid water in a cemented carbide insert, with the internal channels fabricated through electro discharge machining. The non-uniform distribution of the fluid was analysed using computational fluid dynamic (CFD) tools, which indicated a corresponding increase in the cooling rate as the pressure increased. This was observed experimentally on an Inconel 718 workpiece, with reduced tool notch wear and flank wear found on the insert with the internal cooling active. The authors also found a connection between the angular positioning of the internal cooling channel and the flow rate behaviour, which in turn can enhance the heat dissipation.

Shu et al. [20] used a tungsten carbide insert with an insert wall thickness of 1.8 mm and internal wall thickness of 0.7 mm towards the flank face, to spray the cutting zone with liquid water coolant when machining the aluminium alloy 6061. The authors employed a composite turning tool integrated with a pressurised internal and spray cooling mechanism. CFD analysis was performed to ascertain the effectiveness of the design with subsequent testing using a heat flow rate of 10 W/mm^2^ into the insert, and an inlet liquid velocity of 1 m/s at 20 °C. Combining simulation and Taguchi methods, the optimal geometric configuration of the internal channel dimensions was found. By measuring the local temperature with a K-type thermocouple, it was observed that as the tool temperature increased, the spray and internal cooling mechanism were more effective at heat transfer. It should be noted that this method of combining internal and spray cooling is not a closed cooling system, and therefore produces (albeit at much reduced levels), an external coolant to the cutting zone area.

Chen et al. [21] adopted a combined minimum quantity lubrication (MQL) and internal cooling approach machining the nickel based super alloy GH4169. This material exhibits high strength and low thermal conductivity, displaying similar properties to 316L. Using a modified SiAlON cutting tool, an FEM, based on Newtonian cooling interactions, was developed to investigate the temperature distribution field with subsequent validation achieved through machining tests. This design used an internal chamber with two microchannels of the same dimensions, which is fed by a regulated integrated pressurised water based semi-synthetic fluid, with the cooling inlet at the tool end. The first microchannel consisted of a cooling and lubrication dispenser, whereas the function of the second microchannel was primarily to assist chip removal. As expected, the authors found the highest temperature distribution was located at the tool tip, with reduced temperature profile as this region extended radially from the tool-chip zone. It was also found that the region of highest temperature was in the primary and secondary deformation zones, respectively. The results showed an 80 °C drop in tool temperature using the combined MQL and internal cooling system when compared with the dry cutting regime. However, as in Ref. [20], this method used the coolant in combination with the MQL to effectively spray the cutting zone, as such, it was not, strictly speaking, a closed internal cooling system.

Singh and Sharma [22] investigated the temperature variance between dry machining and internal cooling with laminar and turbulent flows models, relating to the cutting tool tip distance from the workpiece depth. The internal cooling system showed a temperature drop of ~ 29% for laminar flow behaviour, and ~ 53% for turbulent flow within the confines of the internal channel. The authors also noted that the temperature cooling effect was more pronounced at the tool tip and this difference radially reduced as the distance from the tool tip increased.

Shu et al. [23] developed a closed internal cooling system in a carbide insert for dry machining using liquid water as the coolant. Finite element analysis found that the optimised thickness of the internal channel walls that could withstand the mechanical loads was 0.1 mm for the rake face and 0.7 mm at the flank face. Applying a heat flux of 20 W/mm^2^, it was shown that the maximum temperature dropped from 381.62 °C to 273.9 °C using an inlet liquid velocity of 0.15 m/s. The authors noted that at higher coolant circulation velocities, the relative effective temperature $\left( {T_{{{\text{eff}}}} - T_{{{\text{max}}}} } \right)/T_{{{\text{ref}}}}$ was the most effective in heat absorption. The results showed the performance of the internal cooling system depends on the geometry of the internal channel along with the velocity and cooling properties of the liquid [23]. These results again indicate internal cooling is an effective means to remove thermal energy in the cutting process, however, it is not stated the material workpiece parameters used in the study.

Isik [24] machined the nickel-based superalloy Waspaloy, using a prototype tool holder with a coated carbide insert. This workpiece material is particularly difficult to machine due to its high shear strength, high chemical affinity, and low thermal conductivity [24]. Employing a 2 mm wide internal channel, with purified liquid water at 18 °C as the cooling agent, the fluid was mechanically pumped in a closed circulatory system with a flow rate of 0.5‒2.0 m^3^/h. Using a pyrometer to measure the average tool temperature over varying cutting speeds, experimental tests indicated dry cutting, with a speed of 95 m/min, produced a temperature of 641 °C. With the liquid coolant applied, there was a reduction in temperature to 587 °C, representing a decrease of 9%. Flank wear was the dominant form of tool wear observed due to the high heat generated, along with the low thermal conductivity of Waspaloy. Overall, the authors indicated a 12% increase in tool longevity using the internal cooling system was achievable. The surface finish was also improved with the internal cooling applied. Using the maximum cutting speed of 95 m/min with a fluidic velocity of 1.6 m/s at a depth of cut of 0.5 mm, produced a surface roughness of $R_{{\text{a}}}$ 0.699 µm [24]. This represents a 13% increase in surface quality compared to the dry machining tests. Again, the highest flow rate and cutting speed produced the best effect in terms of surface quality and heat transfer.

Öztürk et al. [25] used an internal cooling technique to machine 1040 steel and compared the data for tool tip temperature differential and dry machining relating to the average value of the surface roughness. The authors employed an in/out liquid water coolant flow that dispersed onto the base of the insert. Combined with a coolant reservoir, aluminium blocks were used in conjunction with integrated Peltier modules operating as a fan. A CFD model was established and subsequently validated by machining tests. The resultant data showed a 107 °C local temperature drop in the tool tip when compared to the dry test. The measured surface roughness on the 1040 steel obtained a range of values from 0.18 µm to 2.05 µm. Although the results showed a 107 °C drop when compared to dry machining experimentally, it was a relatively complex design.

To ensure good structural conformity in the insert, it is necessary to design the internal channel dimensions within the boundaries of the mechanical reliability parameters. Li et al. [26] used a topological design method to deduce the fluidic behaviour and optimise the structural design of the internal coolant features. The results showed a 180.4 °C drop in temperature using the internal cooling system with the topological design, in comparison to the dry cutting regime.

Ingraci Neto et al. [27] performed experimental machining tests using a prototype cutting tool with a two-phase pump cooling system on 1045 steel during uninterrupted turning. This approach implemented a closed loop design whereby the circulating liquid water vaporised upon contact with a silver interface that acted to cool the cutting tool during machining. It forms with the inlet channel, a tilted annular section of 30 mm^2^ that prevents vapor entrapment and has 106 mm^2^ of heat transfer surface area. The 54 mm^2^ base is in contact with the silver interface. Condensing of the vaporised liquid occurred through forced convection at 25 °C which was stored in an accumulator and then pumped back (via a peristaltic pump) into the internal channel system. The water is pumped at a feed rate of 1.78 min^−1^. Three thermocouples were fixed in contact with the tool to measure the temperature change. The results showed that the temperature of the cutting tool with internal cooling was 79 °C lower than the maximum temperature reached in dry cutting.

Uhlmann and Meier [28] developed a numerical model of the heat transfer mechanism using standardised industrial inserts. In their study, the heat flow is directed orthogonal to the rake face which is then conducted through the tool into a copper heat sink. The accumulated heat is then transferred through forced convection into a dynamic fluid consisting of water/water and glycol flowing parallel to the rake face. The numerical modelling results showed a maximum tool temperature reduction of 21% with the water/water glycol agent relative to water.

This method requires an external pump to drive the circulation of the fluid, a heat exchanger, and a chiller. It therefore is relatively complex compared to more simple designs.

Shu et al. [29] performed numerical and experimental studies on a closed looped internal cooling tool. Using a tungsten carbide insert, modelling showed that a cutting-edge thickness of 1 mm and a wall thickness of 0.7 mm from the flank face, was able to withstand the thermomechanical loading. Thermocouples were used to measure the temperature in the insert during the experiments. Simulation results showed that the maximum temperature reduction of 82.68 °C was achieved with the internal cooling system. Experiments were not conducted in an online machining process, but the authors instead used a modified experimental set-up via heat induction by a solder iron. The results indicate that the effectiveness of the liquid water-cooling system increases as the inlet velocity, heat flux and tool-chip contact area are increased [29].

State of the art review and analysis in internal cooling mechanisms in cutting inserts have revealed the following.

(i) Internal cooling fluids have a positive effect on the heat transfer rate in cutting tools, and this relates to a reduction in tool wear and improved workpiece integrity overall.
(ii) The location and geometrical dimensions of the internal channel has a significant impact on the effectiveness of the cooling system. Furthermore, it can be said that as follows.
  ① There is a direct correlation between the distance of the cooling channel from the main source of heat on the cutting edge, and the effectiveness in heat transfer within the channel.
  ② The location and dimensions of the internal channel can reduce the structural integrity of the cutting insert, therefore considered design of the geometry is required to enable the retention of the structural strength, whilst providing for optimum cooling is paramount in the design process.
  ③ Research suggests that the higher the temperature within the cutting zone, the faster the temperature transfer is achieved when using internal cooling systems.
(iii) The state of the art in internal coolant systems in cutting inserts is primarily centred on liquid water as the coolant fluid of choice. This limits the effectiveness of the fluid in terms of its thermal conductivity.
(iv) To accurately model the thermomechanical effects on the cutting insert it is necessary to employ a combined analysis, this is the basis of conjugate heat transfer. This requires modelling of the solid-liquid phase boundaries.
(v) To date, all the internal cooling methods used a mechanical pump to drive the liquid around the defined inner chamber. There exists no other method that has been successfully implemented in a model or prototype that can achieve cyclic circulation, and removal of the heat currents, without an integrated external power source.
(vi) Based on the current research, it can be extrapolated that the use of liquid metals as an internal cooling substance in cutting inserts is novel. Furthermore, the use of a magnetohydrodynamic drive integrated into the internal cooling system is original in its proposal.

This work describes the design and fabrication process involved in creating an aluminium oxide cutting tool with an internal cooling channel formed through a ceramic additive manufacturing method. This study aims to investigate the design, analysis and performance of an aluminium oxide cutting insert using a developed thermomechanical numerical model combined with controlled experiments on a custom-made turning machine. Liquid gallium as an internal coolant, combined with permanent magnets to generate a homogeneous magnetic field, forms the basis of a heat transfer mechanism through a magnetohydrodynamic drive. Thus, allowing enhanced heat transfer within the boundaries of the defined geometrical structure of the internal channel, without the need for external coolants or mechanical power input.

Experimental results showed at $V_{{\text{c}}}$ = 250 m/min, the corner wear VB_c_ rate observed was 75 µm with the coolant off, and 48 µm with the coolant on. When increased to $V_{{\text{c}}}$ = 900 m/min, the corner wear VB_c_ rate showed 357 µm with the coolant off, and 246 µm with the coolant on. To provide further validation of the new internal cooling system, experimental tests were compared against the results of the liquid gallium coolant versus external liquid water coolant. At $V_{{\text{c}}}$ = 250 m/min, the difference between the tool wear rate reduction with the internal coolant relative to the external coolant was 29%. Increasing this to $V_{{\text{c}}}$ = 900 m/min, the difference observed between the internal liquid gallium coolant relative to the external coolant was 16%.

## Materials and methods

### Cutting insert design

#### Insert material

For this study, aluminium oxide ceramic material LithaLox 500 (Lithoz GmBh, Austria), containing a high purity (99.99%) slurry suspended in a photopolymer matrix was used as the forming compound shown in Table 1 [30]. The alumina slurry is highly viscous and can form complex geometries accurately via the 3D printing process thus reflecting the original CAD design. This additive manufacturing method is therefore ideal for creating internal features in ceramic materials.

**Table 1:** Physical properties for sintered LithaLox 500 alumina [30]

As sintered	Value
Relative density/%	98.4
Porosity/%	1.6
Purity/%	99.8
Surface roughness $R_{{\text{a}}}$/μm	0.9
Theoretical density/(${\text{g}} \cdot {\text{cm}}^{ - 3}$)	3.985
Hardness [HV10]	1 450
Thermal conductivity/(${\text{W}} \cdot \left( {{\text{m}}\cdot{\text{k}}} \right)^{ - 1}$)	37
Maximum operating temperature/°C	1 650
Specific electrical resistivity/(${\Omega } \cdot {\text{cm}}^{ - 1} )$	~1 014
Relative permittivity	9.8‒10.0
Youngs modulus/GPa	300
Fracture toughness/(MPa·m^1/2^)	4‒5
Four-point bending strength/MPa	430

#### Design of cutting insert

In order to design the cutting tool for its intended purpose, it is necessary to identify the required parameters and conditions during operation. As the intended application is for the ceramic insert to be subjected to significant strain and high thermal load [31], it is necessary to define the machining conditions and associated design parameters as shown in Table 2. By applying these parameters to the material selection and design process, it is possible to achieve a solid foundation on which the fabricated tool can successfully operate under those conditions.

**Table 2:** Physical conditions and design requirements for the ceramic cutting tool [9, 10, 12]

Cutting conditions	Design requirements
High thermal load—chip tool interface	High hardness at high cutting temperature
High stress/strain rate	Mechanical shock resistance
Dry cutting—no coolant	Low wear rate
Continuous—no interrupted cuts	Chemical inertness

**Fig. 1:**
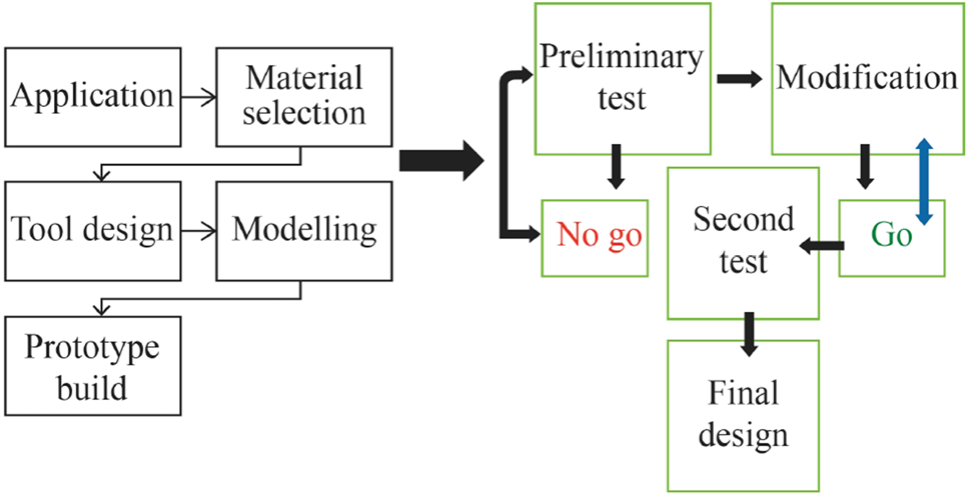
Flowchart illustrating the insert design process used in the study

Applying the design and material requirements in tandem with modelling and physical testing of the initial prototype fabrication ultimately led to the final design. It is useful to visualise this progressive approach by illustrating graphically the method employed as shown diagrammatically in Fig. 1*.* As can be seen, the process itself is cyclic in nature. Once a design criterion is satisfied, the design stage then repeats from that point and then goes through the process sequentially until a further requirement is deemed sufficient to progress. Optimisation of the fulfilled design is then assessed with serial effects considered to the entire tool and machine structure. In this way, the overall design targets are kept at the forefront of the process.

**Fig. 2:**
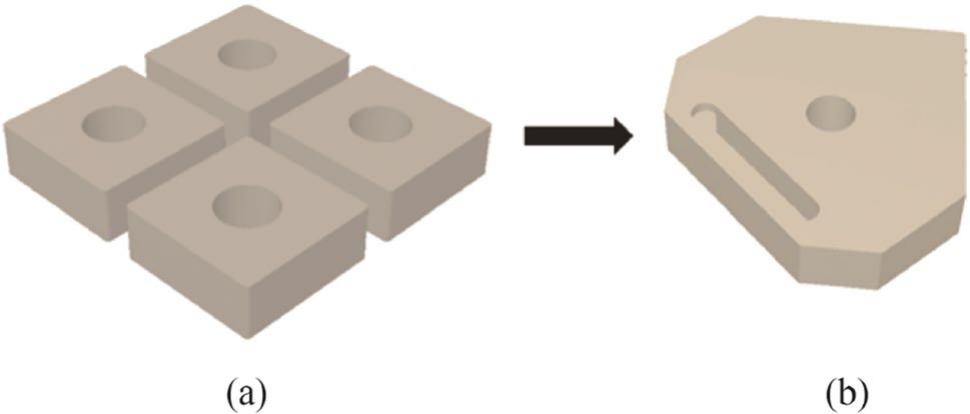
**a** CAD model illustrating the combining of the 4 commercial inserts to form the new model, **b** the final form of the insert

Subsequent to this approach, the insert design was formed by combining four individual commercial ceramic inserts (SNGA 120404) to create a single larger body in the digital design. Physical commercial inserts were not employed, they merely served as a CAD model. This was done to enable traceability in terms of the precise geometric specifications from an industry standard, and then modified accordingly in CAD (see Fig. 2a) with the final model shown in Fig. 2b*.*

#### Design criteria and method for the internal cooling system

Literature review has indicated for effective cooling of the insert the translational distance between the peak heat source and the cooling agent should be within the minimal distance possible whilst maintaining structural integrity. The temperature differential between the peak cutting temperature and the temperature of the cutting fluid should be as high as possible. The cooling agent should have the ability to increase velocity in response to a temperature increase—this follows from previous research that validated the correlation between the liquid cooling velocity and the effectiveness in heat transfer per unit of temperature rise [14]. The cooling agent must also possess excellent thermal conductivity.

**Fig. 3:**
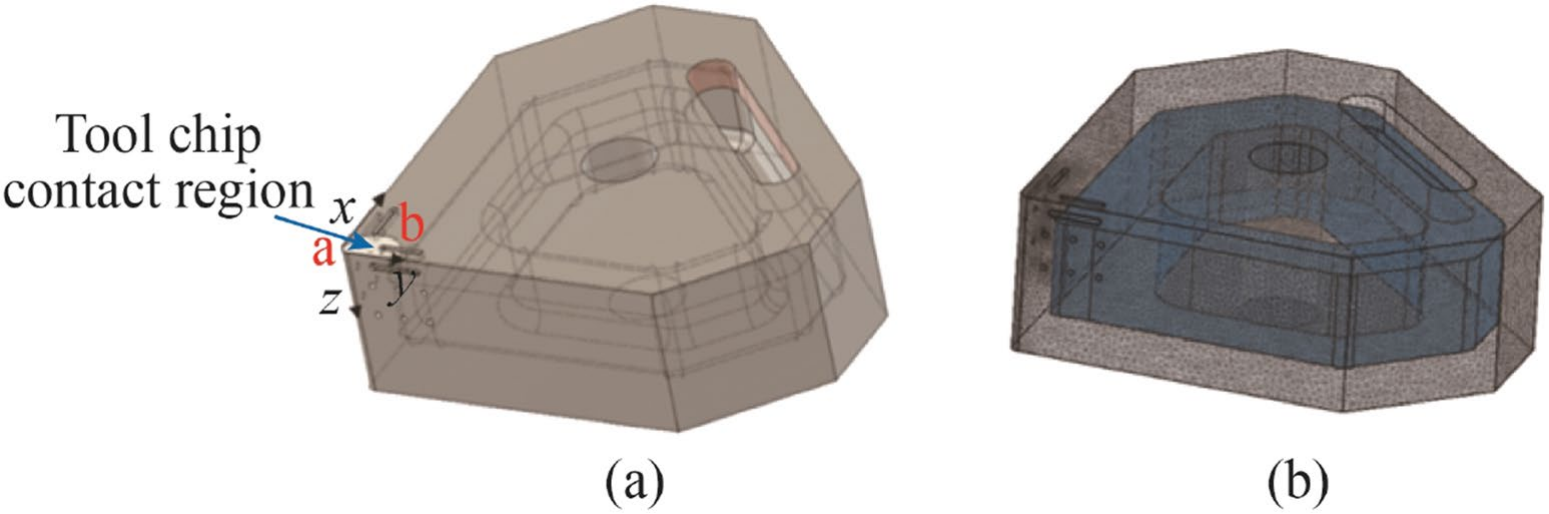
CAD model illustrating the primary tool-chip contact region for heat exchange over the areas a and b **a** with internal channel shown in **b**

**Fig. 4:**
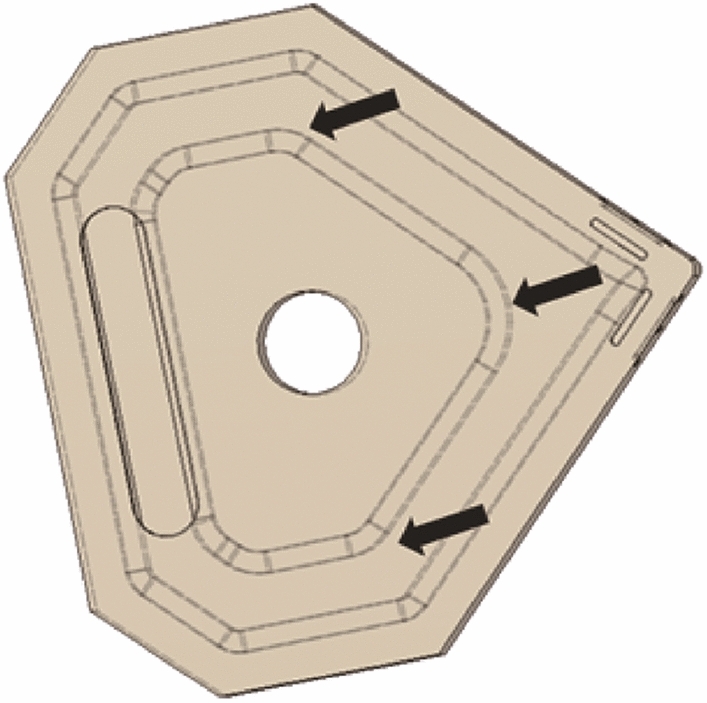
CAD model with arrows highlighting the chamfering of edges during the design stage (this reduces stress concentration points in the internal cooling channel of the ceramic body during densification)

Because the high thermal energy is restricted to the localised region of the cutting insert (see Fig. 3a) the design of the cooling channels necessitates to place it in near proximity to this region (edge, rake, and flank). Therefore, the intended design is to allow for optimal heat transfer whilst maintaining structural integrity of the ceramic tool. A numerical simulation using ANSYS^©^ thermomechanical analysis was used to ascertain the optimum geometrical dimensions (see Figs. 3a, b). Thereafter, the internal cooling channel was selectively chamfered to ensure optimum reduction in stress concentration zones (see Fig. 4).

**Fig. 5:**
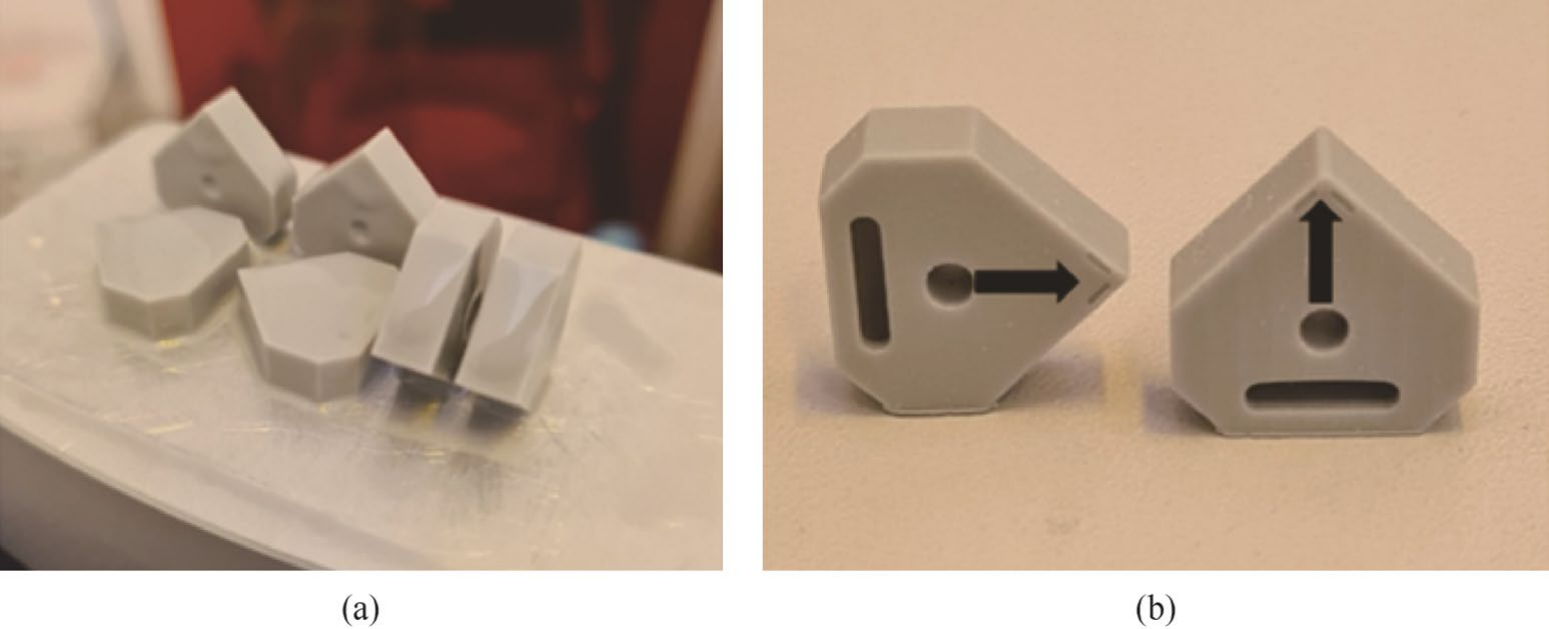
Test parts formed using the SLA resin printer (ASIGA) (Polymer models showing the two orientations used in the test used to validate the orientation selection)

Initial fabrication tests used a polymer material formed using an SLA resin printer (ASIGA) (see Fig. 5a). This was done to rapidly assess the feasibility of the design and thus allow for modification if required. It also was used to examine the orientation of the insert to account for overhangs in the print build (see Fig. 5b), the results of which could then be transferred to the Lithoz digital 3D platform to ensure successful fabrication of the ceramic green body, and thus reduce material waste (and cost) by analysing the polymer model first.

**Fig. 6:**
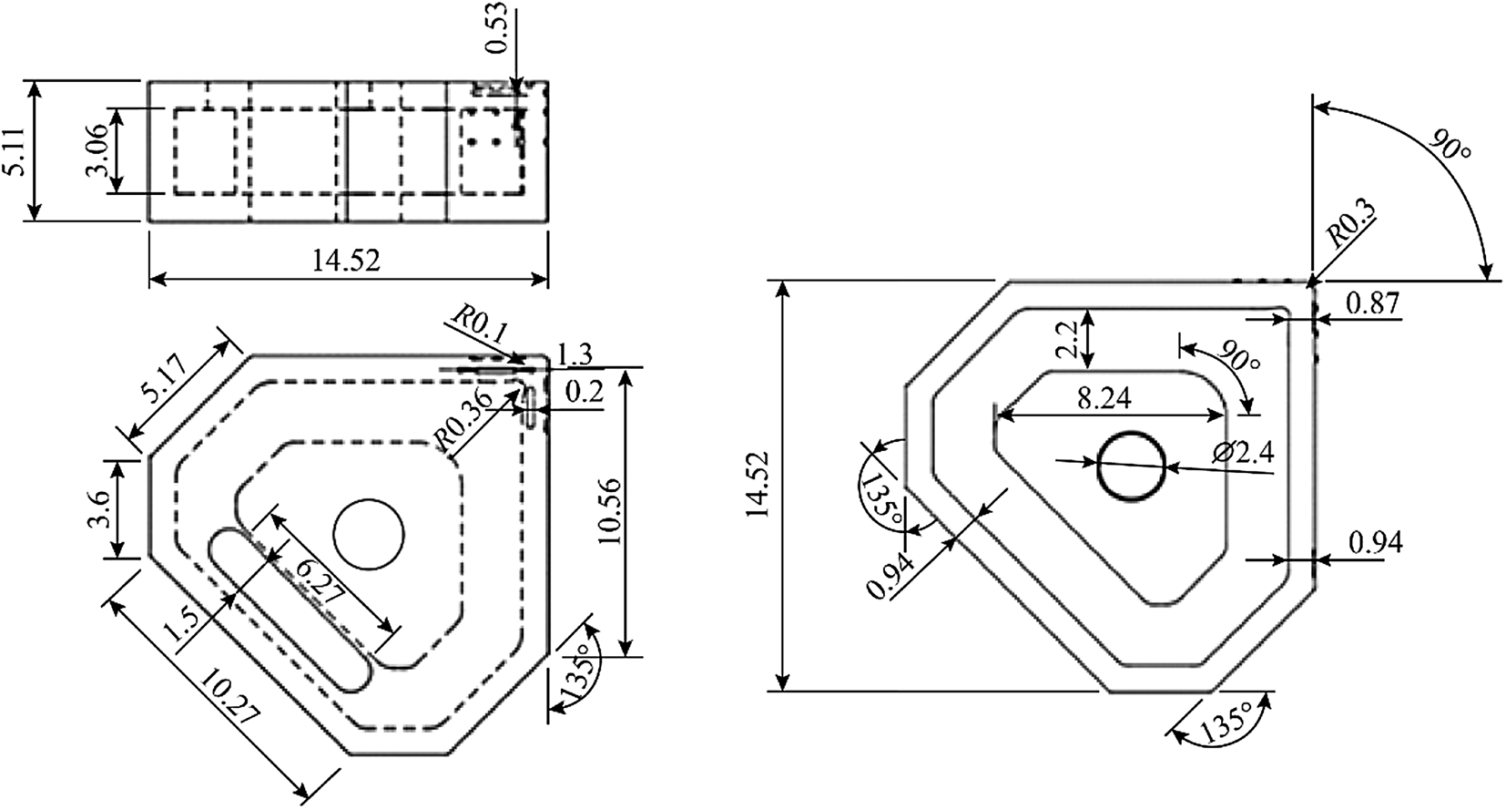
Geometrical specifications of the cutting insert with surface definitions (Unit: mm)

The coupled thermomechanical stress analysis was to ascertain how the design behaved under various loading and thermal conditions to allow structural modifications accordingly and to achieve optimum output in terms of the thermomechanical and geometric configurations. A dynamic thermal distribution of the relevant heat flow into the primary contact regions of the cutting insert was configured. The resultant data were then transferred into the ANSYS interface stress platform for combinatorial evaluation under the conditions shown in Table 3. Several iterations of the structural configuration were conducted until a satisfactory final model was developed. The dimensional and geometric specifications of the cutting insert are shown in Table 4 and Fig. 6, respectively.

**Table 3:** Modelling parameters used in the thermomechanical analysis for the cutting insert

Thermomechanical	Magnitude
*Heat flow/W	25
*Temperature	
Rake edge/°C	600
Major flank edge/°C	500
^1^Loading conditions	
Rake edge/N	700
Major flank edge/N	600

Note: *Source thermographic measurements taken from experimental machining tests, ^1^Source taken from Refs. [6, 7, 12].

**Table 4:** Dimensional specifications of the insert

Geometric definition	Magnitude
Clearance angle major/$\left(^\circ \right)$	5
Insert angle/$\left(^\circ \right)$	90
Rake angle/$\left(^\circ \right)$	0
Cutting edge length/mm	12.7
Circle diameter/mm	2.4
Insert thickness/mm	5.11
Corner radius/mm	0.3
Depth of cut/mm	0.01
Internal channel (depth)/mm	3.06
Internal channel (width)/mm	2.2
Hemispherical surface patterning (diameter)/mm	0.2
Linear patterning (diameter)/mm	0.2
Linear patterning (width)/mm	1.5

#### Surface patterning

**Fig. 7:**
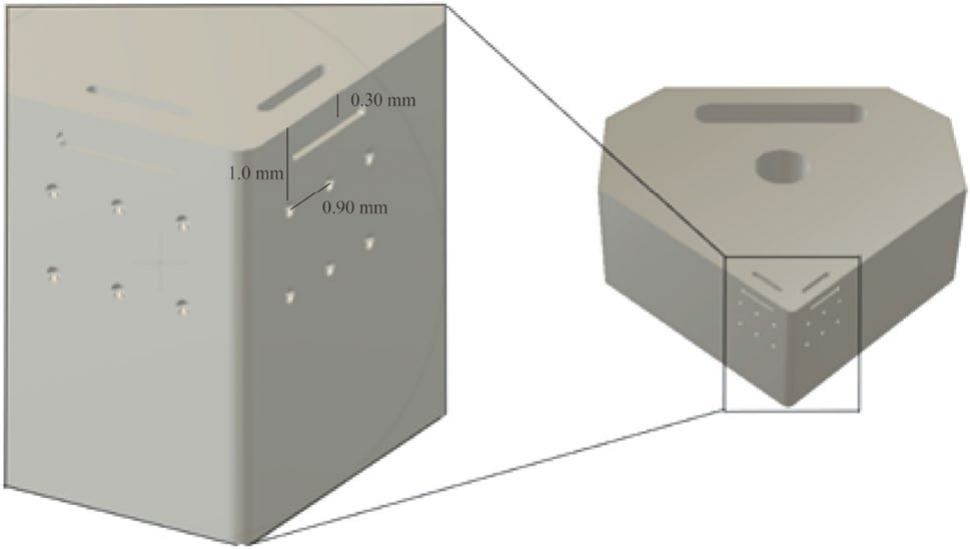
Enlarged region of the insert edge showing the relative positioning of the hemispherical surface patterning (*Ø *= 0.2 mm), and linear patterning (*Ø *= 0.2 mm, *L *= 1.5 mm)

Previous study has shown that modifying the surface of a cutting tool with selective patterning can result in improved wear profile and surface finish [32, 33]. A hemispherical surface patterning was fabricated into the flank and relief faces of the insert. Linear features were also integrated into the rake, flank and relief faces (see Fig. 7). This was done to demonstrate the potential of the AM process only. The functionality of the surface patterning is not included in this study.

#### Thermomechanical modelling

**Fig. 8:**
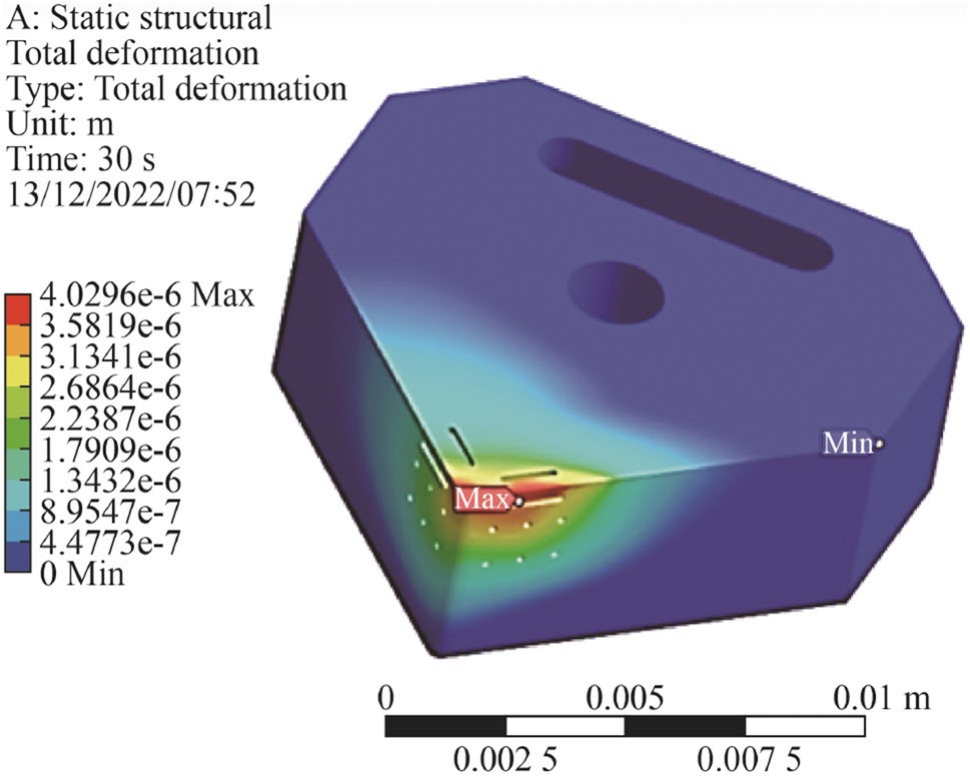
Simulation of structural deformation across the cutting insert

From Fig. 8, it can be seen there is a continuity of stress deformation across the face of the insert and the magnitude is below the structural limitations of the ceramic insert.

**Fig. 9:**
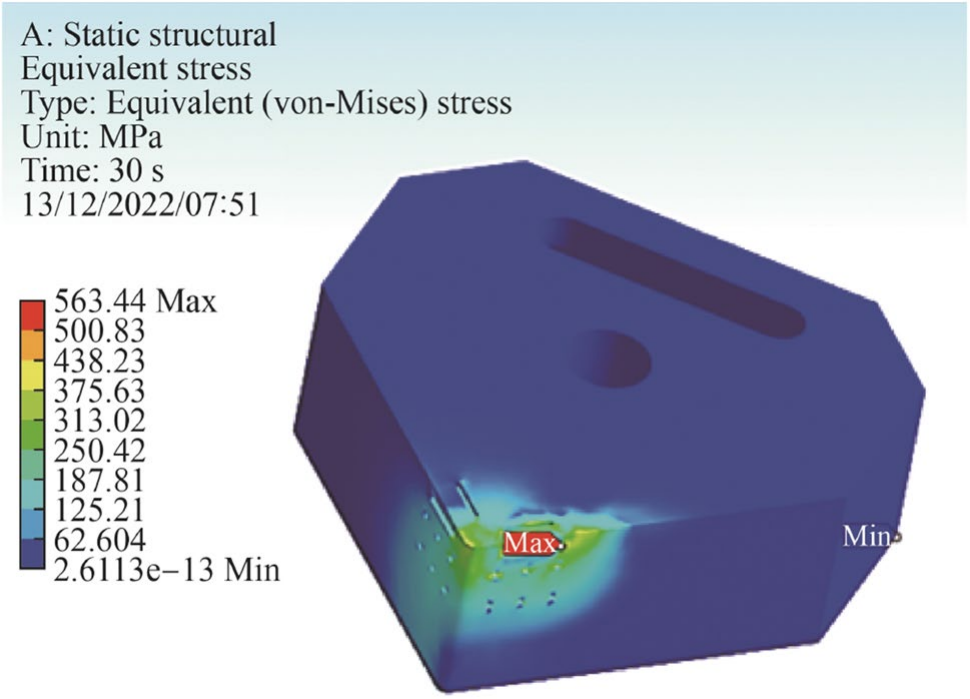
Simulation of stresses (Von Mises) across the cutting insert

For the thermomechanical model, the developed mesh used 25 274 nodes and 15 525 elements. The applied force consisted of 700 N in the *z* direction and 600 N in the *x* direction representing the tangential and radial forces combined [6, 7, 12]. Boundary conditions assumed a fixed support at the central hex nut hole, with secondary supports placed symmetrical at opposite faces on the insert, which reflects the actual physical model. A temperature of 600 °C and 500 °C was applied to the rake edge and major flank edge which reflected the heat experienced by the insert under machining conditions. The maximum deformation was 4.02 µm with an average deformation of 1.06 µm. The minimum temperature was 224 °C and the average was found to be 377 °C. The Von Mises stresses showed a maximum value of 563 MPa with an average value of 83 MPa (see Fig. 9). The magnitude of these indicates that the geometrical design containing the internal channel is sufficient to withstand the applied forces and thermal energy during the machining.

#### Conjugate heat transfer procedure

**Fig. 10:**
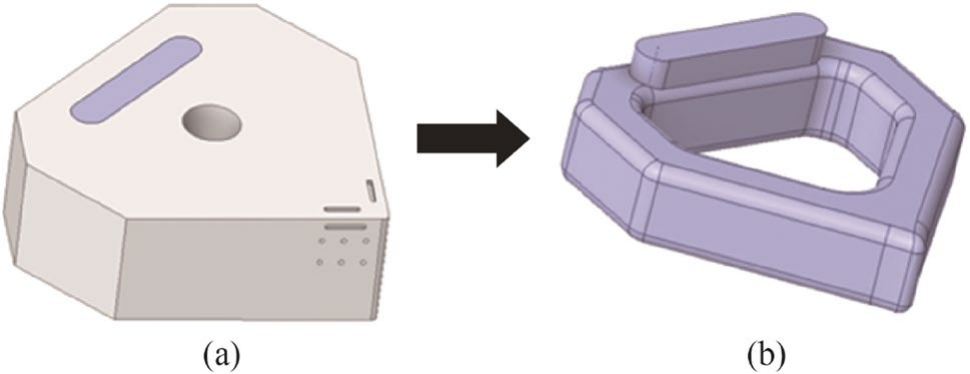
CHT analysis **a** CAD model of the Al_2_O_3_ insert with internal channel, **b** extraction of the fluidic region

The CAD file was directly imported from Fusion 360 into ANSYS FLUENT. It was then prepared for CHT analysis by extracting the predefined inner channel (see Figs. 10a, b). The machining conditions (temperature, heat flow etc.) and material properties used in the thermomechanical model were applied to the insert, with the boundary conditions and solid-fluidic parameters created within the FLUENT tool (see Table 5).

**Table 5:** Simulation parameters for the fluids used in the CHT model (ANSYS database)

Parameter	Liquid water	Liquid gallium
Density (20 °C)/$\left( {{\text{kg}}\cdot{\text{m}}^{ - 3} } \right)$	998.2	5 900
Thermal conductivity (20 °C)/$\left( {{\text{W}}\cdot\left( {{\text{m}}\cdot{\text{K}}} \right)^{ - 1} } \right)$	0.60	37.68
Specific heat capacity/(${\text{J}}/\left( {{\text{kg}}\cdot{\text{K}}} \right)^{ - 1} )$	4 182	373
Viscosity/(Pa·s)	0.001 0	0.010
Reference temperature/°C	20	30
Boiling point/°C	100	2 300

The standard shear-stress-transport (SST) *k*-*ω* model is used. For the tool tip temperature, the value is taken from the three ranges obtained experimentally over the three cutting speeds (see Table 6), the resultant temperatures being 300, 500 and 600 °C, respectively. The applied temperature distribution is radially directed away from the tool cutting edge reducing in temperature according to increasing distance. The ambient air at 20 °C acts as the second fluid boundary with corresponding physical properties incorporated into the model. Two sets of simulation studies are then generated. The first model applies liquid water as the coolant over a period of 20 s with the data extracted from the resultant CHT output results. The second simulation applies the results of the magnetohydrodynamic model and integrates these into the liquid gallium conditions to obtain the results for the subsequent CHT for liquid gallium over 20 s. Both set of data are then formatted accordingly and then compared.

**Table 6:** Machining conditions used for the experimental tests

Parameters	Description		
Machining parameters			
Experiment	1	2	3
Cutting speed $V_{{\text{c}}}$/$\left( {{\text{m}} \cdot {\text{min}}^{ - 1} } \right)$	250	500	900
Feed rate *f*/$\left( {{\text{mm}} \cdot {\text{r}}^{ - 1} } \right)$	0.08	0.08	0.08
Depth of cut $a_{{\text{p}}}$/mm	0.1	0.1	0.1
Machine set-up			
Workpiece	6 mm AISI austenitic stainless steel 316		
Cutting condition	Dry		
Insert material	Al_2_O_3_		
Insert geometric form	Customised		
Turning machine	Customised		

#### Internal cooling channel—fluidic analysis

**Fig. 11:**
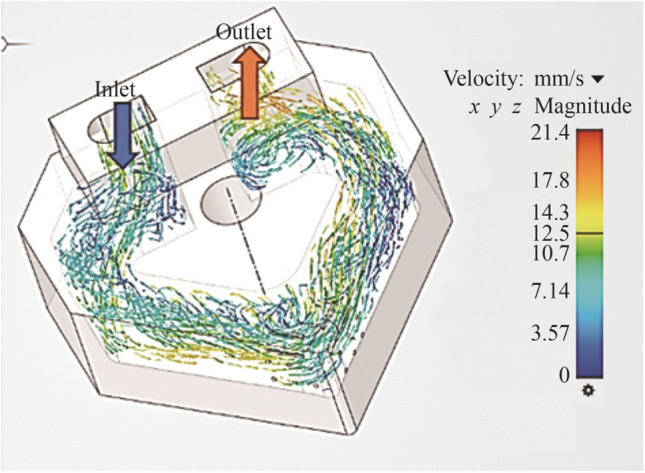
Time lapsed CHT simulation of the internal cooling mechanism (the design reflects the optimum geometrical structure that allows for heat transfer whilst maintaining structural integrity)

FEA simulations using ANSYS FLUENT^©^ were performed to ascertain the optimum dimensional geometry for the tool structure and internal cooling channel. The results of the numerical modelling indicated a structure similar to the initial insert design as shown in Fig. 11*.* These results correspond to the local velocity vectors within the internal channel and the thermal distribution contours in the fluid itself.

#### Magnetohydrodynamic model

To create the magnetohydrodynamic (MHD) simulation, ANSYS Mechanical and FLUENT CFX code employed a laminar flow model through a Reynolds averaged Navier-Stokes simulation (RANS) in the steady state. For the discretization of the domain, a volume mesh computed through the function tool of FLUENT was used. The dissipation and transfer of the generated heat is restricted to the tool, chip, and workpiece of the model.

The temperature through the solid-liquid boundary is transient and depends on the thermomechanical and physical parameters defined in Tables 3–5*.* The simulation used in this study to approximate the solutions of the boundary conditions was solved under an induced magnetic field through a volume of the fluid solver.

The boundary conditions are confined to the geometry of the insert and the internal channel within the solid-liquid boundaries of the material properties assigned to each condition.

To effectively assess the MHD system, it is necessary to integrate the fluid dynamic behavior under the influence of the applied magnetic field. In addition, the thermodynamic results are included in the model for completeness.

Boundary conditions were applied to the solver only for the fluidic region. The external magnetic field *B*_0_ components for the magnetic induction were *x=*0 T, *y = *0 T, and *z = *1.2 T. The solver was limited to the Lorentz force and MHD only, with Joule heating ignored. A transient condition was applied to the solver for a dynamic observation of the fluid behavior at *t *> 0. A viscous laminar behavior was initially applied to the model to reflect the expected velocity rate combined with the liquid metal properties. The simulation used a total of 63 795 nodes and 292 843 elements, with 15 797 nodes and 45 433 elements for the internal fluidic channel.

#### Fluidic velocity—Reynold’s number

**Fig. 12:**
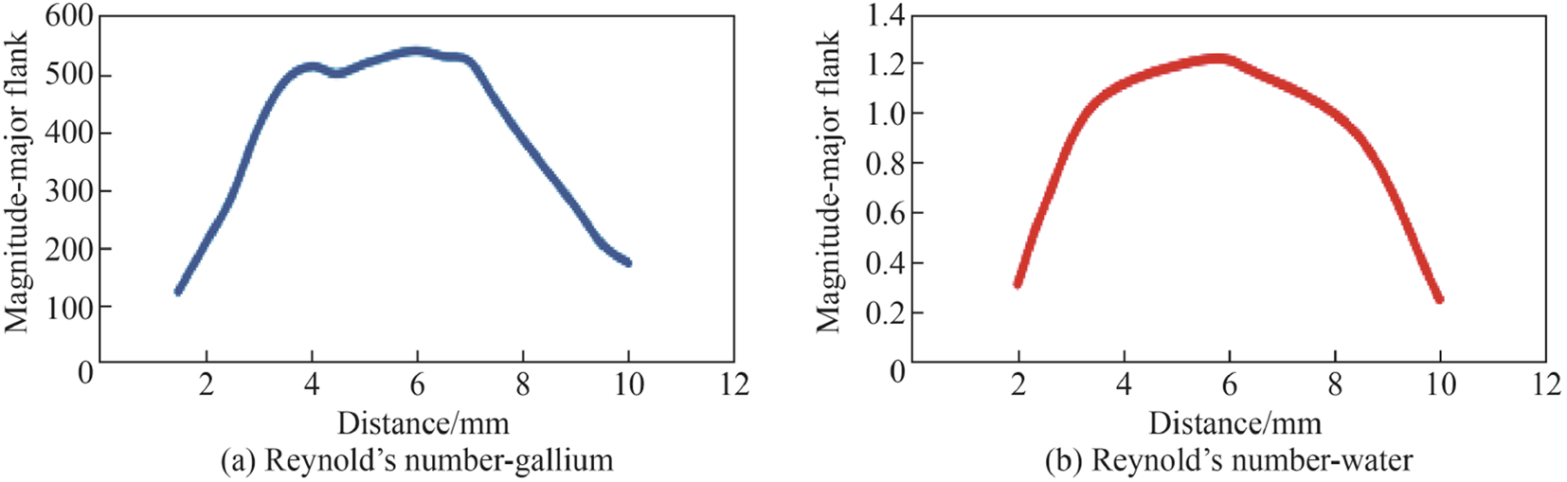
**a** Reynold’s number for liquid gallium in the CFD simulation within the internal channel at the major flank, **b** Reynold’s number for liquid water at the major flank

For the case of liquid gallium, the higher density (relative to water), means that there is a corresponding fluidic restriction within the inner channel dimensions [34]. This is reflected in the increase in pressure. For liquid gallium the peak $R_{{\text{e}}}$ = 565 (see Fig. 12a).

For the case of liquid water, the reduced density (relative to liquid gallium), means that there is a reduction is flow resistance within the inner channel dimensions. This is reflected in the reduction in localised pressure. For liquid water the peak $R_{{\text{e}}}$ = 2.23 (see Fig. 12b).

The increase in Reynold’s number results in an increase in the heat flux flow. The magnitude of the Reynold’s number reflects the type of flow within the internal channel in which it varies from laminar to turbulent. The current set-up indicates a laminar flow behaviour in the channel as the flow behaviour is well below the threshold change to turbulent flow of 2 300 [35]. In terms of the numerical model developed, the internal walls subject to the liquid gallium flow are constrained to the boundary condition of a no-slip interaction, therefore, the fluidic velocity at the boundary is zero. The formulated mathematical model provides a representation of the fluidic behaviour across the entire internal structure. The numerical model developed allows for localised study of regions specific to the geometry under consideration.

### Fabrication of the cutting inserts-lithography-based ceramic manufacturing process

#### Fundamentals

**Fig. 13:**
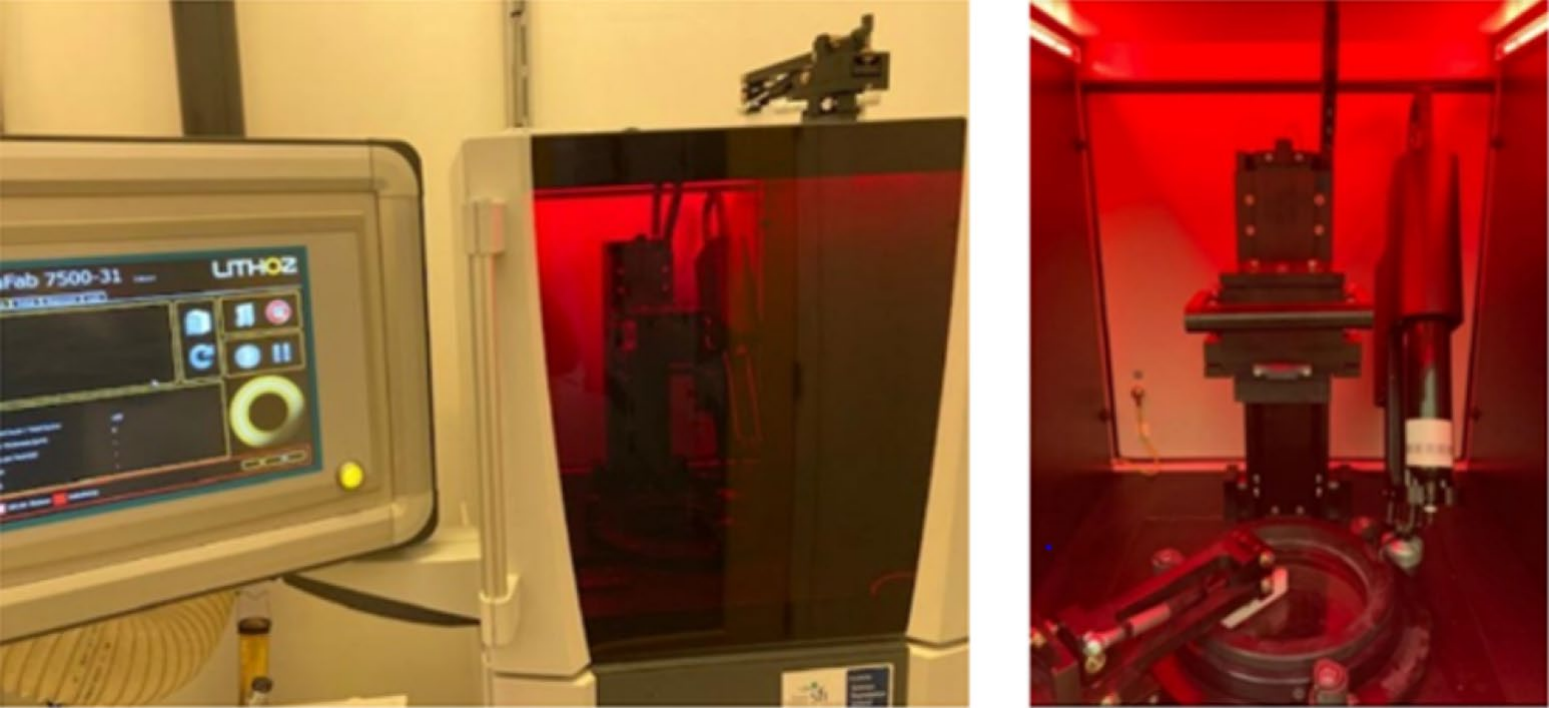
Image of the CeraFab 7 500 and building platform with digital light processing vat

The underlying technology of the lithographic ceramic manufacturing (LCM) process is based on the deposition of homogeneously dispersed ceramic particles suspended in a photopolymer matrix. The resulting ceramic slurry is sequentially deposited in a layer-by layer process that is then subjected to selective curing through exposure to a light source. A digital representation of the model is programmed into the system and contains the geometrical specifications of the part to be formed. Aluminium oxide powders are homogenously mixed in a compound containing a solvent, reactive monomers and a photoiniator. This process, uses light emitting diodes (LED) in conjunction with a digital light projector to produce a 3D structure consisting of ceramic particles, cured sequentially in a photopolymer matrix, which constitutes the green body once formed. LED exposure produces a chemical reaction that results in a methacrylate monomer matrix of suspended ceramic particles representing the original section of the CAD design [36]. For the purposes of this work, a CeraFab 7500 (Lithoz GmbH, Austria) ceramic 3D printer (see Fig. 13) was used to fabricate the inserts. Once this process is completed, the resultant part is the so-called green body which is then removed from the building platform, cleaned with a combination of a solvent (LithaSol 20, Lithoz GmbH) and focused pressurised air. Subsequently, the part was then subjected to a debinding and sintering phase in an air atmosphere for a specified period. After completion of this process the body is now a fully dense ceramic material.

#### Sample preparation

The ceramic parts were fabricated using a high-purity aluminium oxide (99.99%) photocurable ceramic suspension LithaLox HP 500 [37]. This consists of a ceramic based powder blend and a photocurable polymer matrix suspension.

**Fig. 14:**
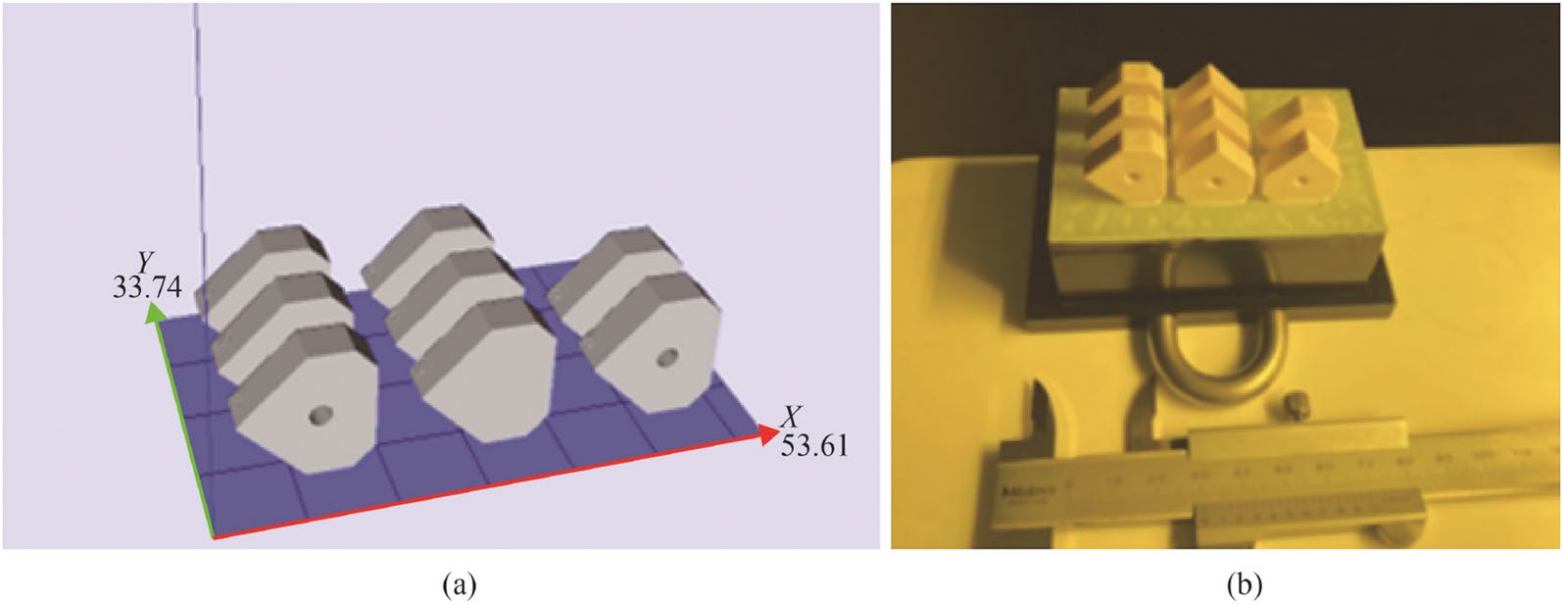
**a** Illustration of the orientations of the CAD models in the virtual building platform of the CeraFab 7 500 DLP machine, and **b** the green body samples after completed build

The CeraFab 7500 uses a standard tessellation language (STL) data format which allows for the CAD model to be transferred to the digital platform for relative positioning as required (see Fig. 14a). The software provides geometrical dimensioning modifications to account for shrinkage during the densification phase. The process used in this study applied a scaling factor of 1.245 to the imported STL files to compensate for shrinkage during sintering. Additional processing parameters (including layer thickness, light intensity, exposure time) are part of the forming phase whereby the LCM software provides for optimised outputs in terms of rheology and dispersion rates of the photocurable matrix thus affecting the overall quality of the ceramic green body [1]. This study employed a 25 µm layer thickness. Formed green bodies are shown in Fig. 14b*.*

#### Debinding and sintering

**Fig. 15:**
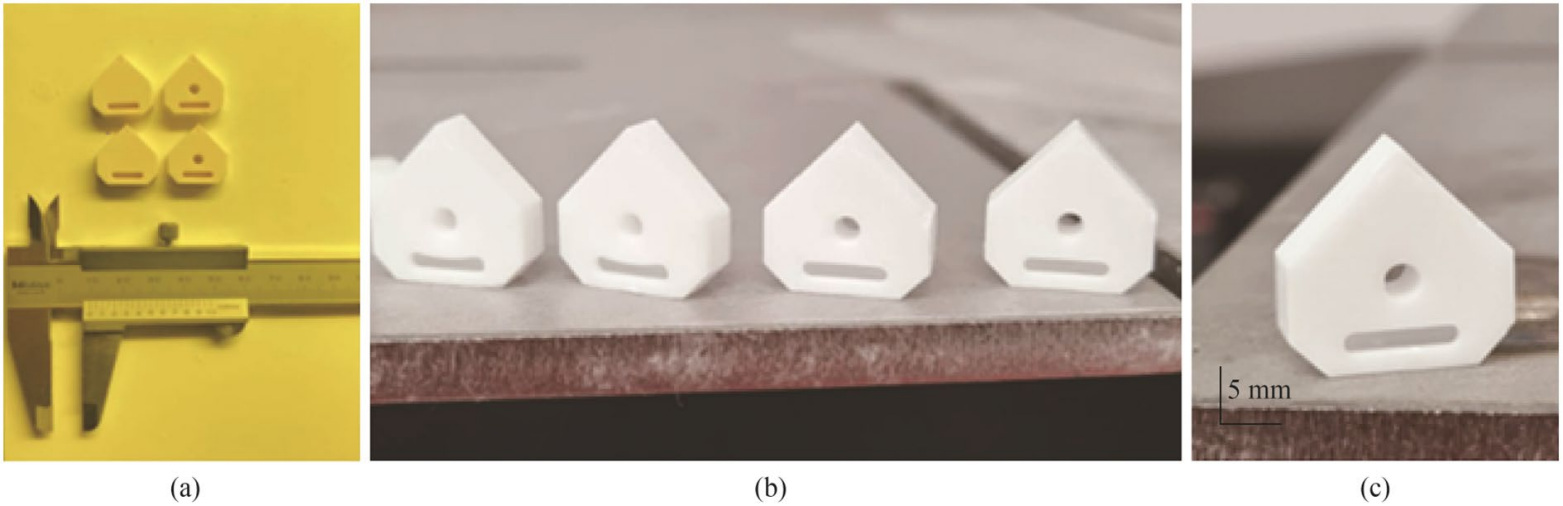
**a** Green bodies, **b** and **c** aluminium oxide sintered parts

**Fig. 16:**
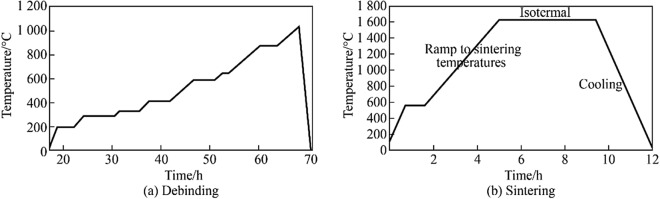
Temperature profile of aluminium oxide **a** debinding, **b** sintering

On completion of the forming and cleaning of the parts phase, debinding was performed in a programable electric furnace (Carbolite Gero RHF 1600) followed by subsequent sintering in an air atmosphere, using recommended parameters from the manufacturer [37]. The green bodies, densified parts and the temperature profiles of the debinding and sintering regimes are shown in Figs. 15 and 16, respectively.

#### Characterisation and sample testing

**Fig. 17:**
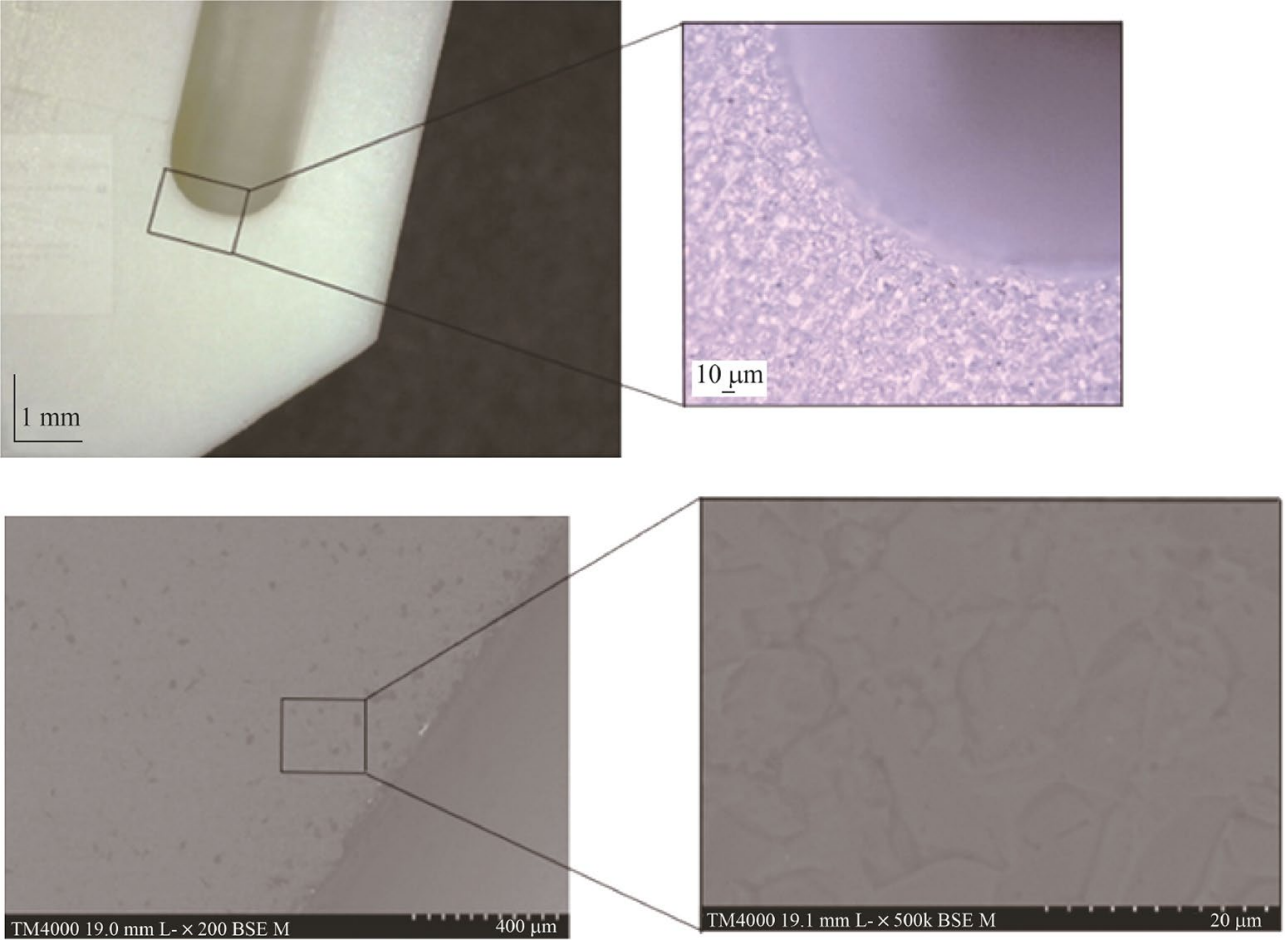
Optical and SEM images of the Al_2_O_3_ insert at different magnifications

The microstructure of the sintered aluminium oxide parts was inspected using a Hitachi TM4000 Plus scanning electron microscope (SEM). Optical images of the sintered parts were taken using a digital microscope (Dino-Lite Pro), and a Keyence 5000 image analysis microscope examined for structural anomalies. SEM analysis was performed on fractured unpolished sintered parts. A sputter coater (Emitech K575X) applied a thin layer of gold to enhance the electrical conductivity prior to imaging. Sintered samples were inspected to identify surface defects as shown in Fig. 17. The results showed good structural consistency in terms of microstructure. There was also evidence of grooves, voids and microfractures in some of the sintered bodies.

#### Lapping and polishing of the ceramic surface

**Fig. 18:**
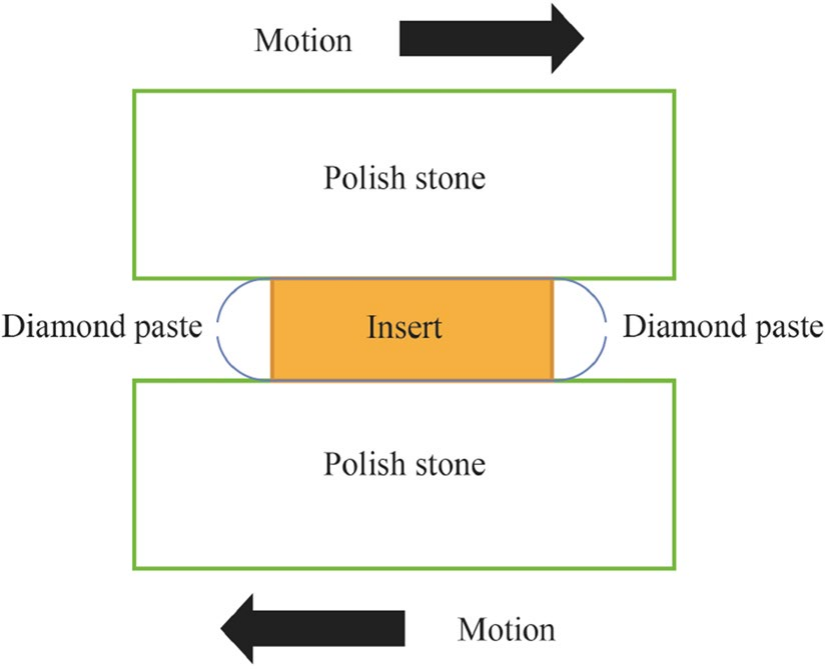
Diagrammatic showing the method employed for the polishing of the ceramic body

To improve the signature surface of the cutting tool which is transferred to the workpiece, a grinding and lapping machine using a series of diamond compounds (Diprofil^©^) from 20, 10, 5 and 1 µm was used. The machine performed 10 cycles with gradual improvement in surface finish (see Fig. 18).

## Results

### Analysis of the cutting insert through the fabrication method

#### Density and strength tests

Prior to testing, dimensional analysis (taken at $20^\circ \pm 0.5^\circ$) was performed on each sample with the average value taken as a reference measurement. Density measurements on selected Al_2_O_3_ sintered parts were carried out using the Archimedes’ method. The results indicated values of 3.933 g/cm^3^, which corresponded to ~ 99.3% of the theoretical density according to available data on Al_2_O_3_ [10]. Strength testing using the four-point bending method (DIN EN 843-1) on samples that exhibited no fracture lines revealed 410 MPa with a Weibull modulus of 10.8 (DIN EN 843-5). Both of these test results are in good agreement with literature on aluminium oxide material properties [10].

#### Internal structure: micro X-ray CT scans

In order to inspect and analyse the internal structure of the sintered parts, a non-destructive micro X-ray computed tomography (CT) system (Nikon XTH225 ST) was employed. This technique allows for volumetric observation of the internal and external features of the printed part. This provides for rapid identification of voids, cracks and discontinuities. However, it is not yet widely used as a method of dimensional metrology relative to established industrial techniques [38].

**Fig. 19:**
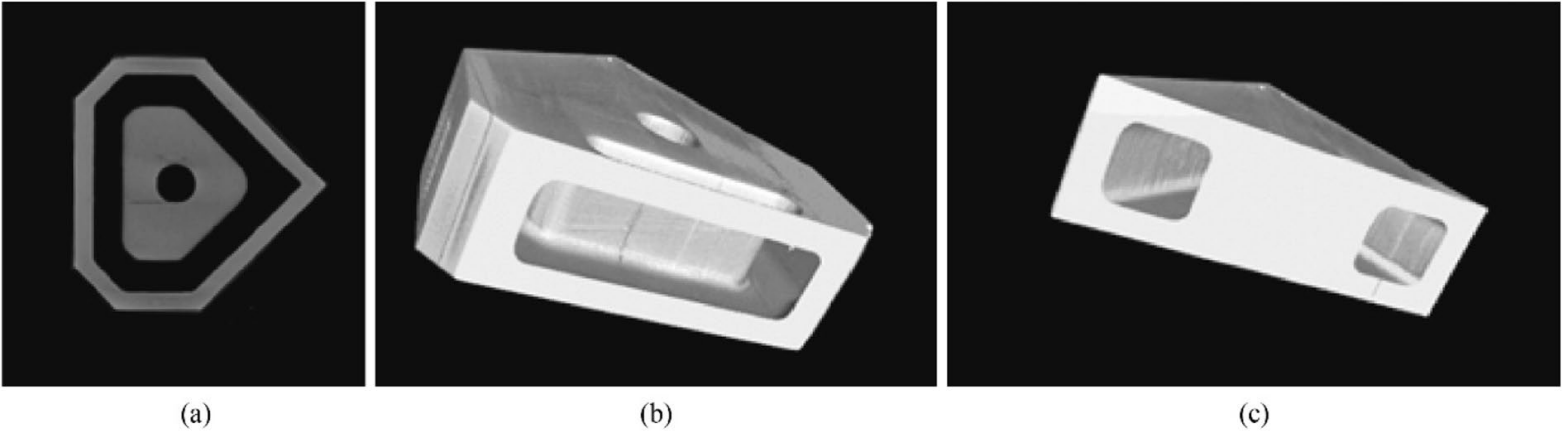
**a** Digitised CT image slice with CAD geometry overlapped for comparison (light green), **b**, **c**, time lapsed dynamic 3D CT image of insert

In Fig. 19a, a digitised scan of the insert is shown with the original CAD model (in green) superimposed on the structure for comparison. It can be seen there is a good geometrical correspondence between the original design and CAD model of the 3D printed insert. In the same Figs. 19b, c, are freeze frame images of two-time phases of the µCT scan; this provides illumination on the internal structure not visible through other means.

**Fig. 20:**
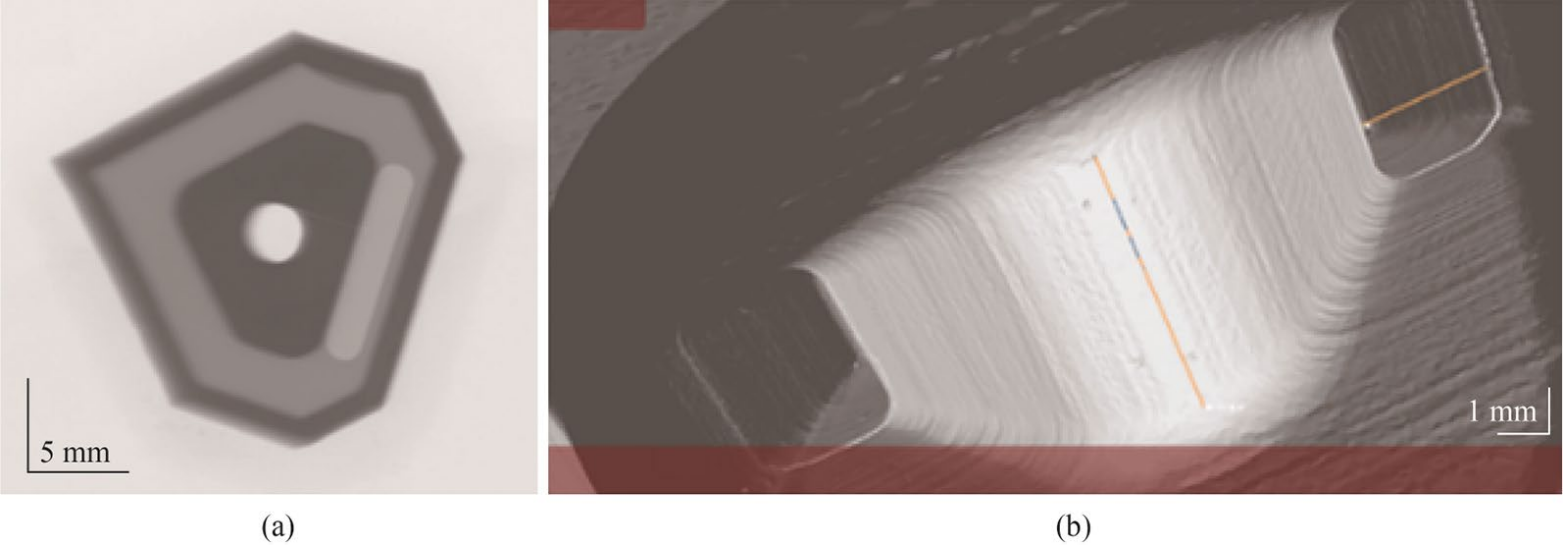
**a** X-ray, and **b** CT image of the internal channel

Figure 20a shows an X-ray image of the internal channel with Fig. 19b providing greater details on the internal structure of the insert post densification. It can be observed that the internal feature has good structural conformity throughout the central region. This was confirmed by taking measurements of the vertical and horizontal components of the inner chamber dimensions relative to the original design. For the vertical component there was a lateral deviation of ±15 µm and the horizontal component revealed lateral deviation of ±23 µm.

**Fig. 21:**
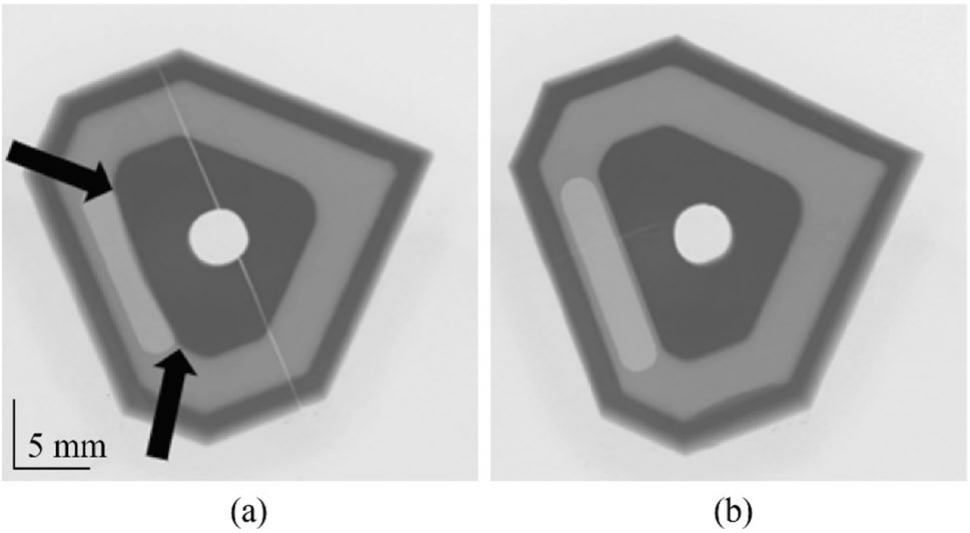
Micro X-ray images showing **a** a linear fracture present in sample and **b** not observed in sample (the arrows highlight the bulging effect reflecting the different orientation used in the LCM process)

Fractures were observed in some of the sintered samples. In Fig. 21a, there is a clear linear crack extending across approximately the centreline of the body, crossing the central hole. This sample was made during the initial stages of forming and heat treatment. In the same image, there is also an overhang feature represented by a physical bulging of the material. This was caused by the orientation of the material during deposition in the build formation stage. This was not observed in the sample of Fig. 21b*,* which was formed using an upright geometry relative to the fractured sample.

#### Surface roughness

In this study (0.84 ± 0.05) µm in $R_{{\text{a}}}$ (average value taken over 6 samples) was found. This slight variation reflects the variation in process parameters used during forming and also the sintering regime. Additionally, other factors such as the mechanical parts and conditions of the components in the building platform contribute to the quality of the part produced. This was evidenced when a glass disc at the base of the CeraFab 7500 vat was replaced: the improvement in surface quality was distinct.

### Analysis of the insert under experimental conditions

#### Experimental tests using a customised turning machine tool

To test and validate the formed insert under controlled conditions, a custom-built desktop sized turning machine was developed. This allowed for comparison of the Al_2_O_3_ insert containing the internal coolant, against the same insert additively manufactured and tested under the same machining conditions.

#### Procedure

Preliminary tests are performed on the machine tool to assess the functionality of the internal cooling system. Experimental tests are performed using the aluminium oxide ceramic insert on commercially available 6 mm stainless steel 316L, under dry and internal cooling machining conditions on the physical prototype machine tool. Table 6 shows the machining parameters applied during the tests. For the test runs, pre-test checks on tool run-out were evaluated using a dial test indicator. A thermographic camera (FLIR TG297) is used to measure the heat transfer. The camera emissivity was set by taking the known emissivity of high purity Al_2_O_3_ [39] and then changing the camera setting accordingly. To ensure accuracy in the measurements, the camera was calibrated by the manufacturer (FLIR) in advance for the specific purpose indicated.

#### Results: tool heat transfer

**Fig. 22:**
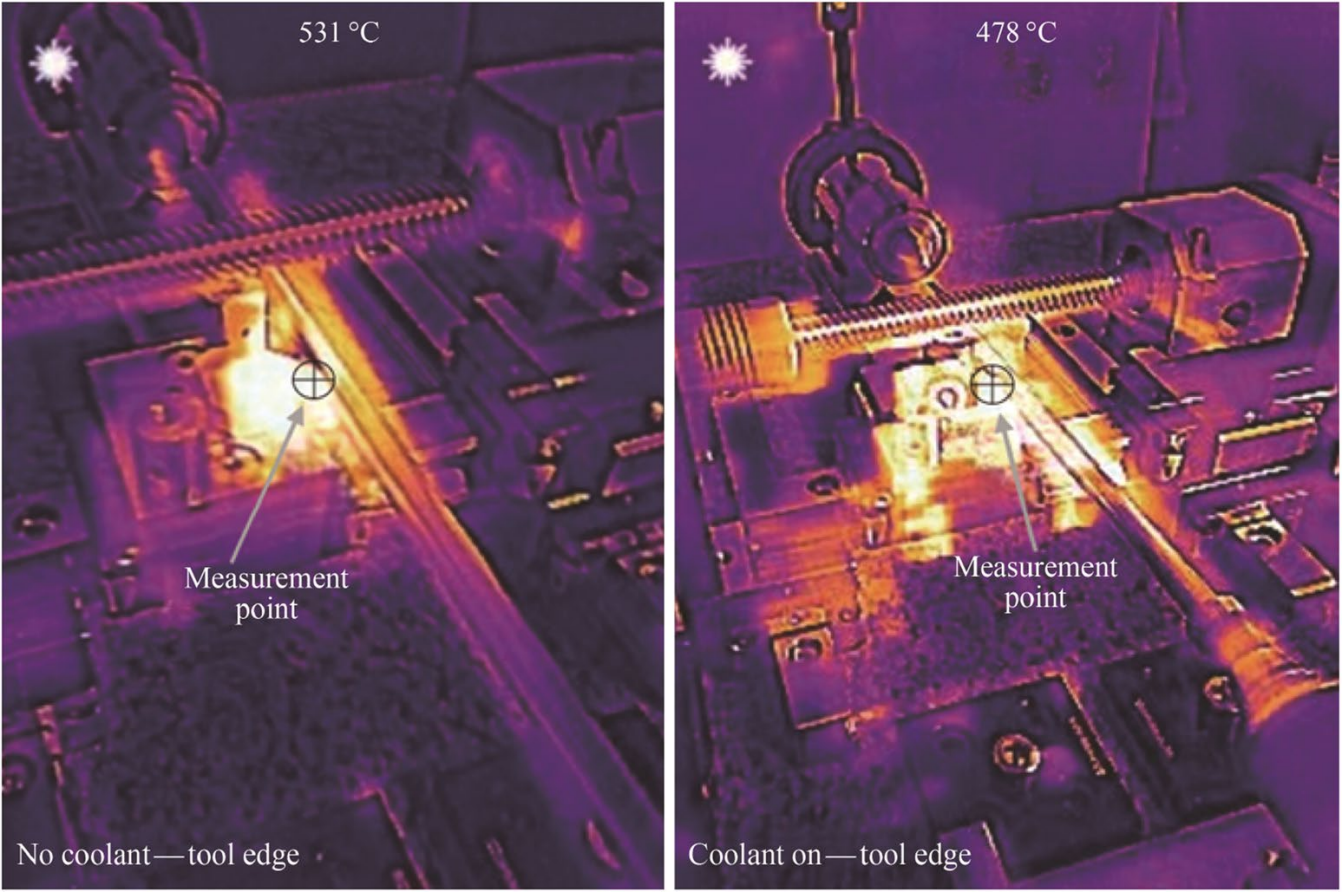
Thermographic images of the heat transfer for no coolant tool edge and coolant on tool edge at $V_{{\text{c}}}$ = 900 ${\text{m}}/{\text{min}}$

For the cutting edge temperature measurement (see Fig. 22), it can be seen that there is a clear variation in the temperature readings obtained with the cooling on (478 °C) and off (531 °C). The difference records a magnitude of 53 °C. This indicates that the internal cooling is working to transfer heat through the tool much more effectively than the monolithic aluminium oxide tool itself. It is clear from the images that thermal energy plumes are more prominent in the surrounding structure of the machine tool with the internal coolant active. This can be accounted for through the larger heat energy dissipation through the local zone of the cutting tool system, which is radiated out into the immediate surroundings.

There is a general correlation between increasing the cutting speed and tool wear which results in higher temperatures and faster tool wear. Moreover, the cutting speed itself has the largest effect on the cutting temperature [9]. Additionally, along with the cutting speed, other factors such as the cutting tool condition (microfractures and excess wear of the flank) can lead to increased temperatures in the cutting zone [9]. The interdependency of this relationship allows for thermal imaging of the cutting zone to provide data on the magnitude of heat generated. The thermographic method to measure the tool-workpiece interaction is through positioning the thermal imager in such a way that it can record in-situ the dynamic cutting zone during the machining process [9]. However, this is not at all easy to do in practice. The extreme conditions in the vicinity of the cutting tool along with the motion of the tool and workpiece when machining, mean the actual cutting process can be obscured from target view. In this study, a series of measurements is taken using different positioning locations and angles of the camera to attempt to capture the best location.

#### Tool wear

The degree of tool wear directly impacts the surface roughness of the workpiece. In fact, the signature of the tool is transferred into the surface of the workpiece. Thus, as tool wear progresses, then in turn, the surface integrity is reduced. One of the main causes of excessive tool wear is large amounts of thermal energy which occurs in uninterrupted cutting such as turning operations. The optimisation of suitable cutting parameters, the cutting time and tool wear rates are important considerations in establishing an accurate measurements of surface roughness [9]. However, this section on experimental validation of the cutting tool is primarily concerned with the magnitude of heat transfer and corresponding tool wear that occurs using the internal cooling system. Therefore, it is not within the scope of this study to include machining variables which can modify the resultant rate of tool wear or surface roughness. To do that would be prohibitively time consuming and add detailed complexity to identifying what is effectively a heat transfer problem under controlled conditions. In light of this, the cutting time, cutting parameters (depth of cut, feed rate) are kept constant, apart from the cutting speed as previously stated. Also, for each measurement, one cycle of 100 mm cutting distance was used.

**Fig. 23:**
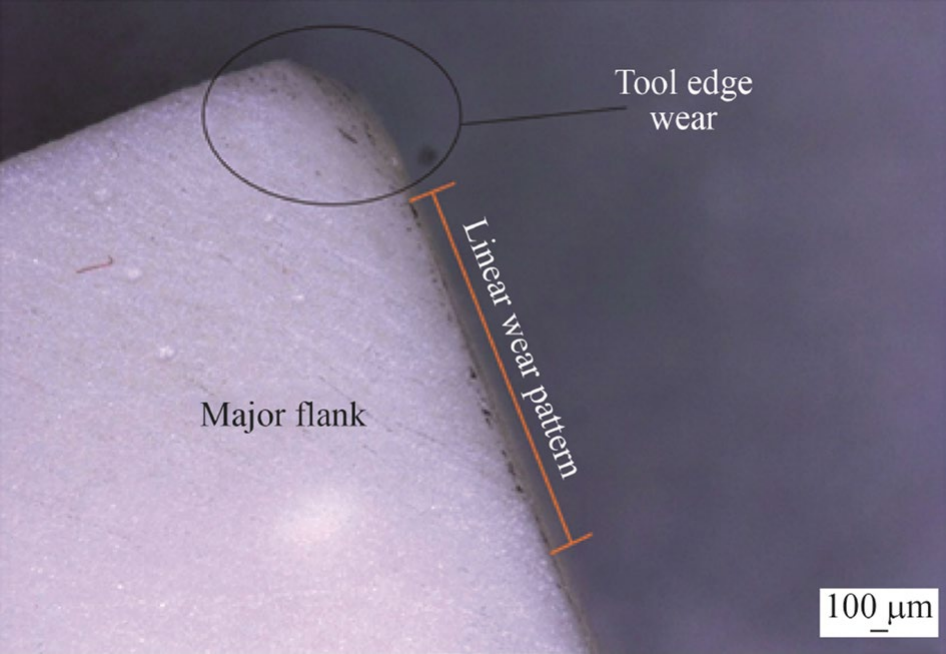
Optical image of the tool wear profile for Al_2_O_3_ insert with MHD cooling system on for 10 cycles

For both cases (cooling on/off), a new insert was used during each of the machining tests relative to the various cutting conditions employed. Observationally, it was found that there was a relationship between the magnitude and geometry of flank wear at lower cutting speeds of 250 ${\text{m}}/{\text{min}}$. Excessive wear patterns were obtained which showed an unfavourable association between the Al_2_O_3_ insert and the lower cutting speed using these machining parameters. Figure 23 shows the types of wear patterns observed during the dry machining of the 316L workpiece under machining speed $V_{1}$ after 10 cycles.

## Discussion

The quality of the densified parts depends mainly on the processing parameters during the forming phase, and the debinding and sintering regime [1]. During the set-up stage of the forming process, the CeraFab 7500 allows for variations in parameters such as layer thickness, light intensity and exposure time. Any changes to one of these would invariably affect the photoreactivity of the suspended slurry, which in turn effects the end component. However, correctly applied forming parameters are some of the advantages of the LCM process, which can produce superior feature resolution and similar or better surface finish density (> 99%) of the sintered parts with the benefit of geometrical range potential over conventional fabrication methods [1, 5].

### Mechanical tests

**Fig. 24:**
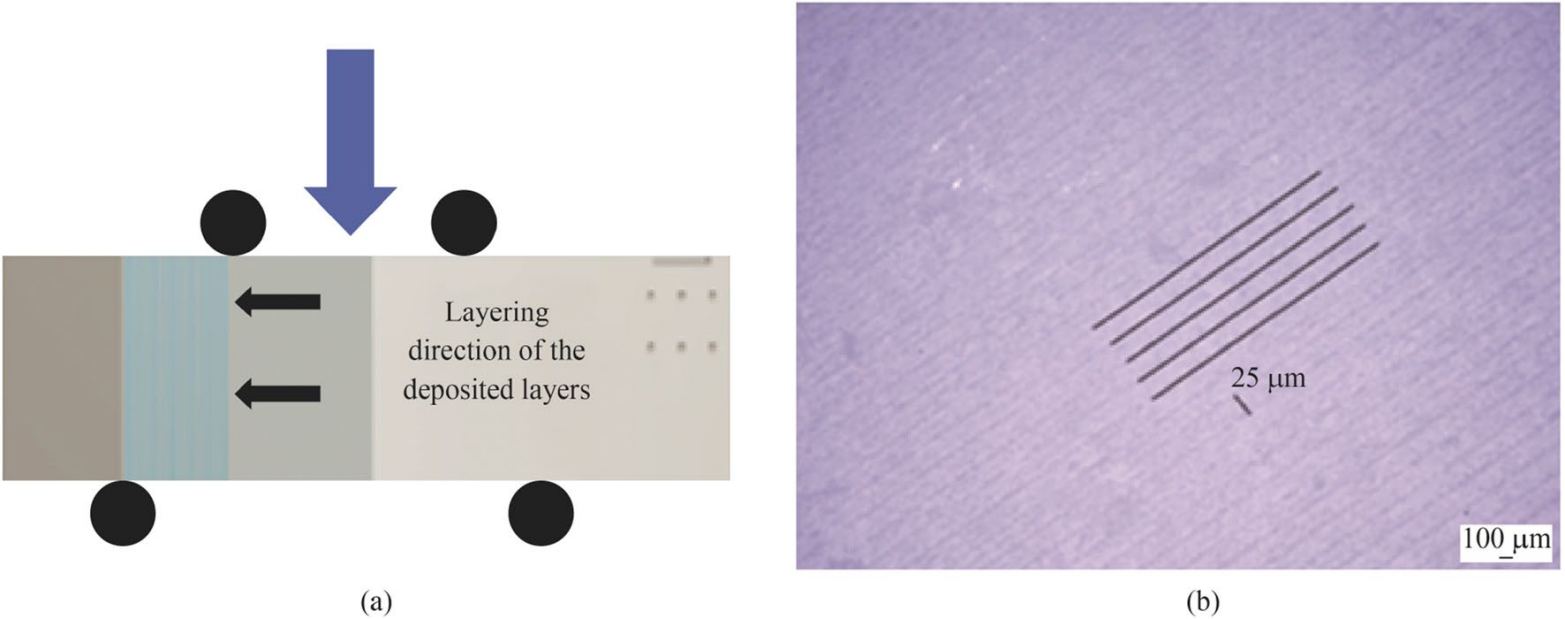
**a** Diagrammatic illustrating the direction of the mechanical tests relative to the layered deposition, **b** false colour optical image of the layer thickness in an Al_2_O_3_ sintered part (the parallel lines correspond to the deposited layer thickness (25 µm))

During mechanical inspection, the geometrical orientation of the test parts was fixed with the set up shown in Fig. 24a. In this diagram, the mechanical strength was measured using the sequentially deposited boundary layer as the point of examination. This approach was employed previously by Schwentenwein and Homa [1]*,* as it preferentially tested the theoretical weakest region according to the layering structure of the LCM process. There was some variation in terms of Weibull modulus values collected. This may be due to surface variations/defects which contributed to a lower value in some cases [40]. The thickness of the deposited layers can be seen in false colour (see Fig. 24b).

### Digital build forming

**Fig. 25:**
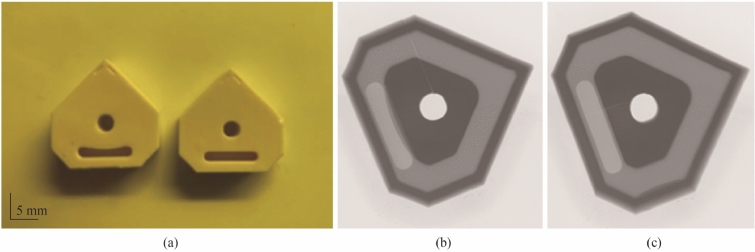
**a** Comparison of green bodies with the deformation displayed, **b**, **c** X-ray images of sintered inserts highlighting the geometrical deviation in **b**

As previously noted in the section of results (see Fig. 21), deformation of the densified insert was seen with bulging present after scanning. This was also observed in the forming phase of the process. In Fig. 25a, there is a distinct bulging in the sample on the left, which is attributed to the method of layered deposition during the forming phase. This is further seen in Fig. 25b when compared to Fig. 25c for comparison. The CeraFab 7500 allows for the CAD model to be placed in a predetermined spatial geometry according to the desired method in which the layers are deposited and subsequently cured by LED light. In this case, the insert design has an internal feature that is significant enough to warrant consideration on the optimal orientation of the build platform. Analysis of the overlap and internal features were conducted prior to the selection of the build orientation, and it was deemed to be useful to perform the build on two different orientations to provide insight into the forming process.

### Optical and SEM analysis

Visual inspection showed defects at different regions of the sintered parts on various samples tested. This was attributed in part to the cleansing technique post forming. In this method a cleaning solvent (Lithosol) was selectively directed at excess material left over from the LCM process. A controllable pressurised spray filled with the solvent was applied to the surface of the green body. During this process, the sample is typically held in the operators’ gloved hands housed in a plastic shielded enclosure containing the pressurised solvent spray. Mishandling, over focusing of the jet spray on the surface and accidental microchipping occurred when performing this initially. Proficiency in the technique improved over time. It was suggested to use an ultrasonic bath cleansing method to improve the process quality [36]. A modification on this approach may improve the consistency in part production which extends to the quality control process.

**Fig. 26:**
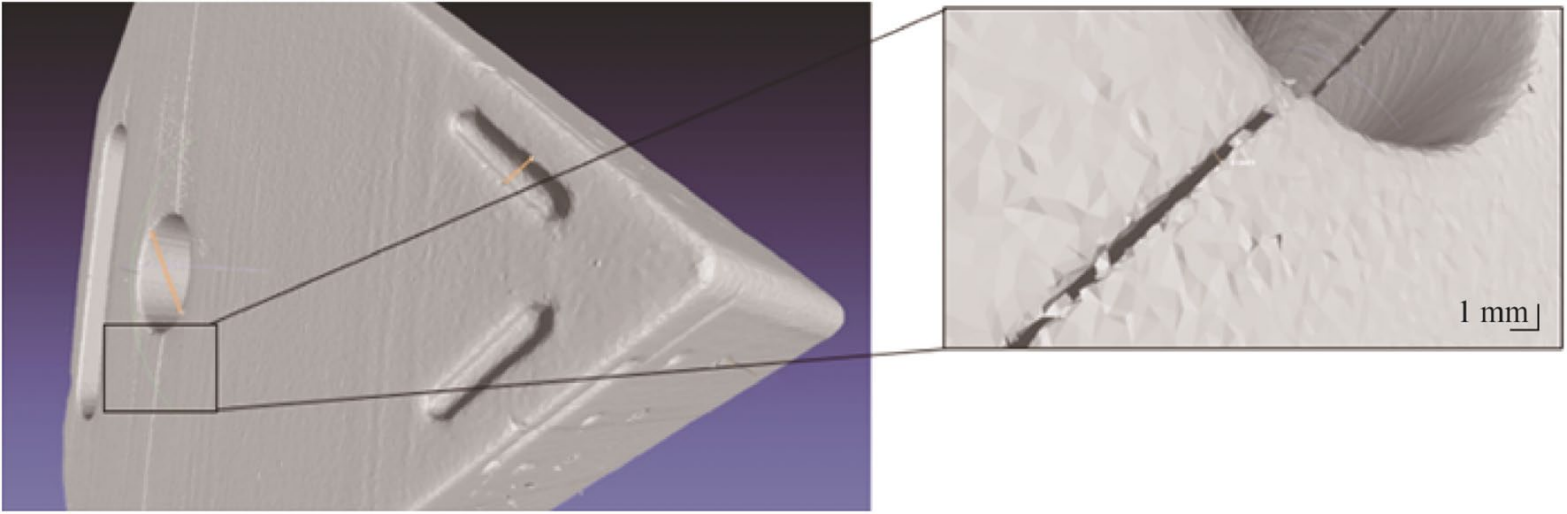
µCT image of full linear fracture with enlarged region shown (width of fracture 416 µm)

Although the fracture lines visible in the optical and X-ray images appear to have a preferential direction under inspection (see Figs. 21 and 26). With the internal channel geometry and heat exchange hole initially attributed as the source of the fracture growth in terms of structural design. However, changing the orientation of the green body pre sintering showed that the movement of the alumina particles during densification differed depending on the geometrical positioning relative to the local heat inside the furnace chamber. It is thought that this reflects the way thermal energy is transferred to the green body and subsequent ceramic microstructure during the growth phase. The fractures are likely caused by an excess heating regime. Lowering the heat rate incrementally produced parts with less fracture generation. Further testing on the heat treatment phase showed that when the thermal regime was experimentally modified, the resultant part did not display any significant fracture lines. It is believed that the growth of the fracture was likely due to the forming process and rapid heat treatment.

### Microstructure

The microstructure of some samples showed uniform distribution of the Al_2_O_3_ particles with no obvious sign of voids or fractures (see Fig. 17). The resultant conclusion from the images suggested even distribution of the ceramic slurry and by extension, this rheological behaviour is also present in the deposition process. As the microstructure reflects the mechanical strength of the sintered parts [41], any change in the sintering driving force will in turn modify the end result produced. Likewise, modification of the debinding and sintering stage will affect the mechanism and degree of removal of the binder matrix and the coalesce process associated with the grain boundary movement.

The STL geometries were compared against the sintered parts in µX-CT analysis (see Fig. 19a). The results indicate very minor spatial variations in terms of dimensional accuracy which gives confidence in the LCM process. The LCM and subsequent heat treatment phase should be viewed holistically as part of a whole process as opposed to two different stages in the AM method. This is because of the strong interconnection between the forming and sintering process and the microstructural morphology produced after densification. Optimising the specific digital design directly influences macroporous morphology, which in turn impacts the thermodynamic properties of the ceramic structure [42]. Therefore, these two processes are strongly linked, and this should be at the forefront of design considerations from inception.

### Layering effect

Due to the nature of the LCM process, which uses a predetermined layering parameter that produces deposition of the slurry material, surface roughness measurements can spatially vary. Therefore, an average value (0.84 ± 0.05) µm in $R_{{\text{a}}}$ was taken over six samples directed parallel to the boundary layers which invariably exhibited the least smooth surface.

The layering effect remained on the outer regions of the sample after densification. Again, this was due to the manufacturing method which employs sequential deposition of defined layer thickness. However, a sample was fractured to inspect the inner structure surface and this layering effect was not present. The lack of layering indicates the action of the thermal treatment which results in good bonding during densification.

### Sintering

In the sintering process, the green body is heated below its melting temperature, for Al_2_O_3_, the melting temperature is 2 073 °C corresponding to a peak value of 1 600 °C applied in the heat treatment process. Instead of the compacted body melting, densification (porosity reduction) occurs via atomic diffusion (movement across lattice zones) [43]. In this study, the heating regime used a constant rate of temperature increase until an isothermal temperature was reached (1 600 °C) and held there for a dwell period of 2 h. This was followed by gradual cooling at room temperature to avoid thermal shock. From a manufacturing design perspective, in terms of producing the required physiochemical properties associated with alumina, the type of microstructure required is first identified (e.g., small grain size for increased toughness) and the process parameters, rheology and heating regime are then decided based on the defined microstructural requirements [43].

The mechanism of grain growth is a complex process. Summarily, grain boundaries are regions of lattice disorder within the microstructure, where each grain/crystal is separated through a lattice mismatch [40]. Typically, grain growth occurs in conjunction with pore growth (coarsening). The decrease in free energy in conjunction with the reduction in the grain boundary area is the driving force for grain growth, given as $\Delta E_{{\text{g}}} \approx - A_{{\text{s}}} \gamma_{{{\text{gb}}}} /2$, where $\gamma_{{{\text{gb}}}}$ is the grain boundary energy.

**Fig. 27:**
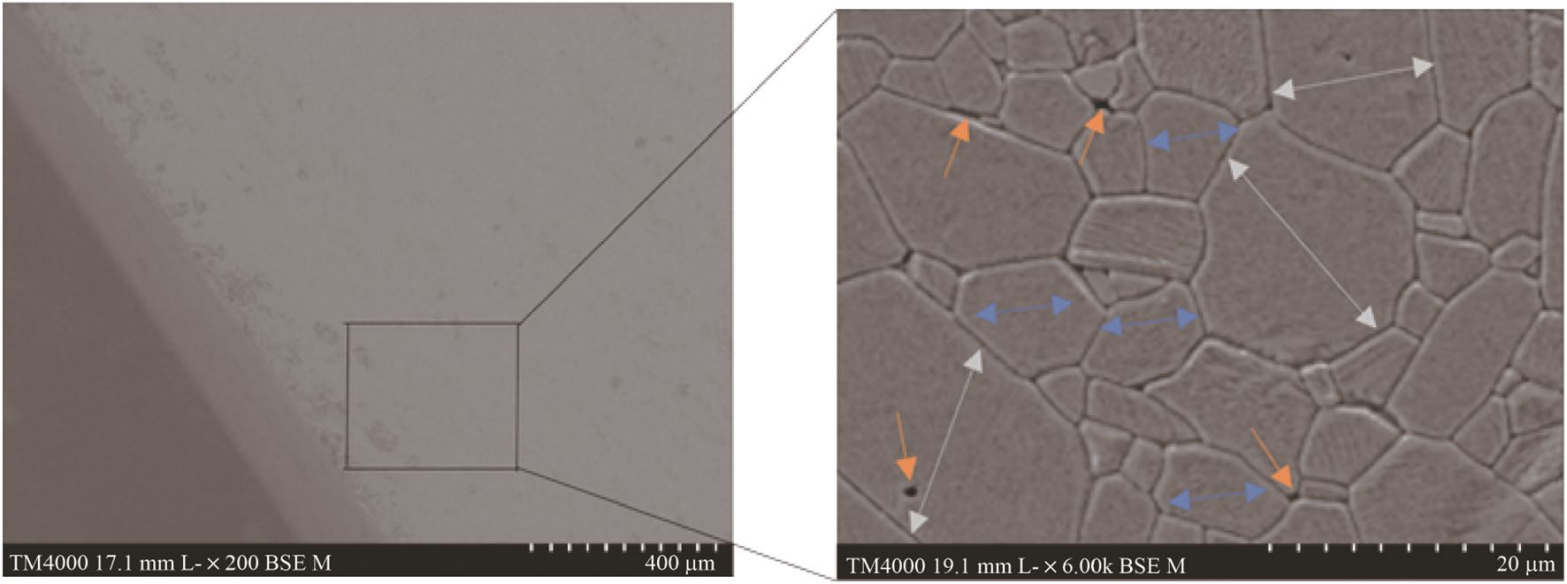
SEM illustrating normal and abnormal grain growth in Al_2_O_3_ (the grey arrows show regions of excess growth distribution, whereas the blue arrows show normal grain growth. The yellow arrows show regions where voids were observed)

This can be normal or abnormal grain growth. Figure 27 shows an SEM image of a sintered part exhibiting structural inhomogeneities (packing density and pore size variation). The presence of these inhomogeneities can lead to a reduction in the densification rate producing voids during the sintering phase. The same image also shows that the average grain dimension and shape are similar within a given area (blue arrows), this is normal grain growth distribution. However, there are regions displaying overgrowth where extended growth occurs relative to the localised matrix. This type of microstructural behaviour is characterised by abnormal growth. Higher sintering temperatures results in quicker densification but produces increased coarsening [43]. This in turn can lead to abnormal grain growth, affecting the final density of the sintered body.

The control of grain growth is important because it directly affects the material properties of the ceramic, and by extension its suitability for a desired application. Therefore, tailored density design in the forming microstructure requires control of the growth kinetics in sintering. From an engineering materials viewpoint, it is also worth noting that creep resistance increases with grain size, conversely, fracture toughness increases with smaller grain size [25].

The irreversible sintering process produces a reduction in the free energy of the system (the sintering driving force) which is defined by particle curvature, external pressure and the chemical reactions.

The heat treatment throughout the tests used a programmable electric furnace in an air atmosphere. Different results were obtained depending on the debinding and heating parameters used. This was the case for all samples manufactured and thus reflected the importance of the sintering regime.

### Fracture of parts

Internal fracture occurred in some samples post densification. These likely are attributed to a combination of process parameters and thermal stress [44]. Highlighted fractures are the result of liquid film formation on the grain boundaries (directionally dependent) in the heat affected zone along with internal tensile stress. Residual stress is another factor that affects the generation of microfractures in the ceramic structure. This can be controlled through judicious selection and optimisation of the part design, layer thickness and process parameters specific to the design requirements with heat treatment optimisation [44]. Taking this further, the CAD design should reflect the manufacturing method proposed, thus enabling identification of the manufacturing challenges before the forming process begins. Based on this approach, the entire design process needs to consider the manufacturing route in which the design is conceived and adjusted accordingly.

### Porosity and densification

Modification to the sintering dwell time may offer optimisation of the grain size and associated densification of the ceramic body [45]. Highlighted research that suggested changing the temperature and dwell time to a two-step phase can yield fine grain microstructure and complete densification with maximum fracture toughness. Aside for temperature control and heating schedules, particle size of the green body (affecting densification rate), size distribution, shape and form of the particle also contribute to sintering outcomes [43, 46, 47]. This is the case regardless of the manufacturing method employed (conventional or AM processes).

### Tool heat transfer effectiveness through the cooling channel

Although the internal cooling mechanism can address the dimensional restrictions of the tool size and associated heat capacity of the Al_2_O_3_ body, it remains problematic regarding the thermal conductivity of the ceramic material. Aluminium oxide has a relatively low thermal conductivity, and this limits the effectives of the internal heat removal mechanism. To address this, study is being conducted on a novel method using liquid gallium as an enhanced mechanism to transfer internal heat, this forms the basis for the subsequent section on performance analysis of the internal cooling tool.

#### Magnitude of tool wear reduction: dry versus MHD cooling

**Fig. 28:**
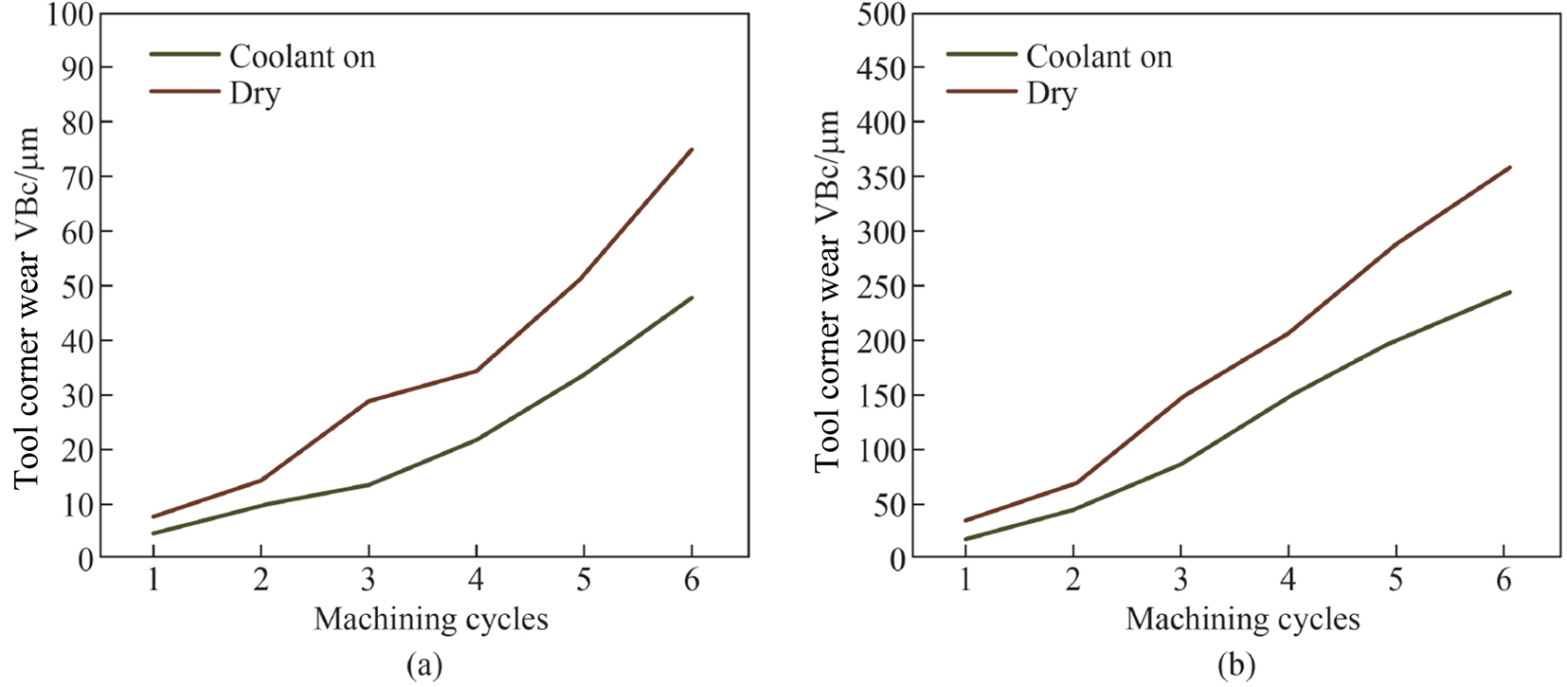
Corner wear VB_c_ rate corresponding to number of machining cycles for the cutting insert under conditions of dry and cooling at **a** $V_{{\text{c}}}$ = 250 ${\text{m}}/{\text{min}}$, and **b** $V_{{\text{c}}}$ = 900 ${\text{m}} / {\text{min}}$

Figure 28 shows the percentage of tool wear reduction at two cutting speeds over a period of six machining cycles. The results indicate that at the lower cutting speed $V_{{\text{c}}}$ = 250 ${\text{m}}/{\text{min}}$, the corner wear VB_c_ rate recorded 75 µm with the coolant off, and 48 µm with the coolant on. The difference between the tool wear rate with coolant active relative to no coolant is 36%. When the cutting speed was increased to $V_{{\text{c}}}$ = 900 ${\text{m}} / {\text{min}}$, the corner wear VB_c_ rate observed was 357 µm with the coolant off, and 246 µm with the coolant on. The difference between the tool wear rate with coolant active relative to no coolant is 31%. Therefore, tool wear reduction is achieved using the internal coolant.

#### Magnitude of tool wear reduction: MHD cooling versus external cooling

Using an external coolant (liquid water) as an alternative comparison using a motorised pump which fed water through a malleable conduit at 2.5 ${\text{mL}}/ {\text{s}}$ at an ambient temperature of 20 °C.

**Fig. 29:**
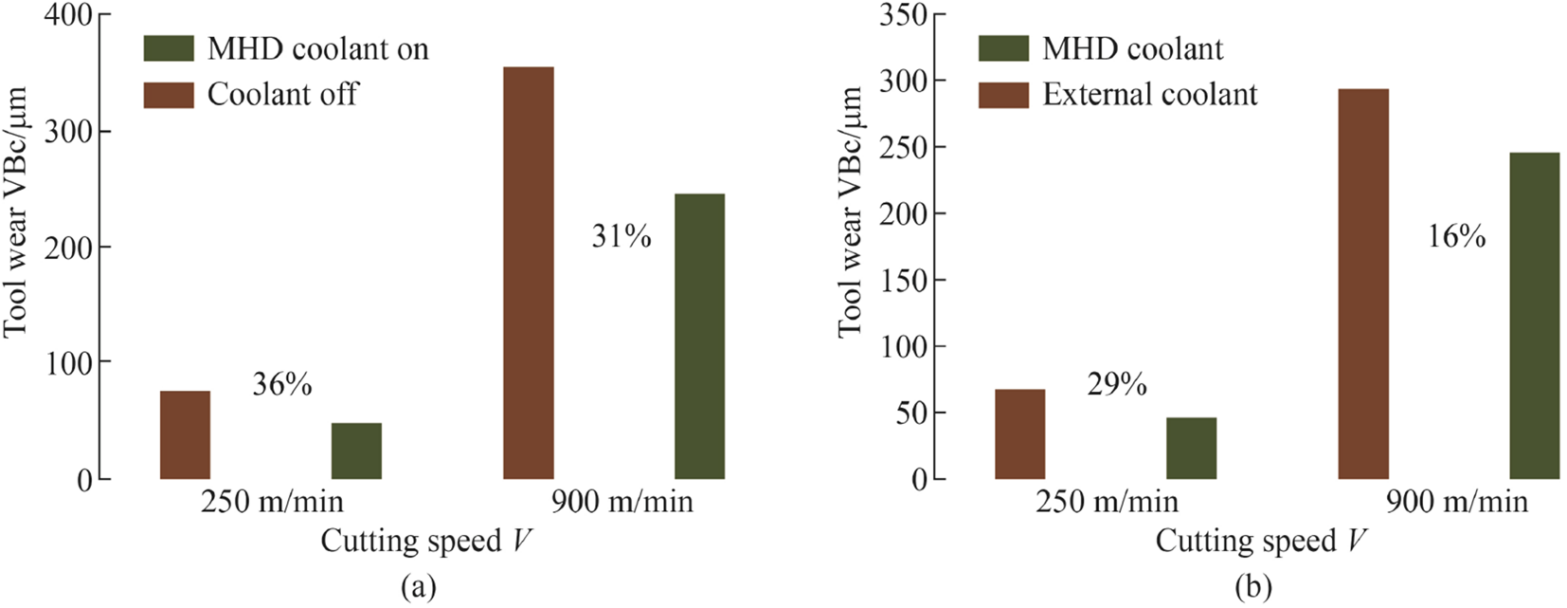
Tool wear effects under two machining speeds with the corresponding percentage difference in wear shown with the cooling on and off (tool wear effects of the MHD cooling system vs external cooling with the corresponding percentage difference in wear shown between liquid gallium (internal) and liquid water (external))

The resultant wear profile of the two inserts are shown in Fig. 29. Using the same cutting conditions as previously indicated, the corner wear VB_c_ rate at the lower speed observed was 68 µm with the external coolant, and 48 µm with the MHD coolant. This represents a decrease of 29% in tool wear difference. When the cutting speed was increased to the higher speed, the corner wear VB_c_ rate showed 294 µm with the external coolant, and 246 µm with the MHD coolant. The difference between the tool wear rate reduction with the MHD coolant relative to the external coolant being 16%.

#### Heat transfer in the internal channel

**Fig. 30:**
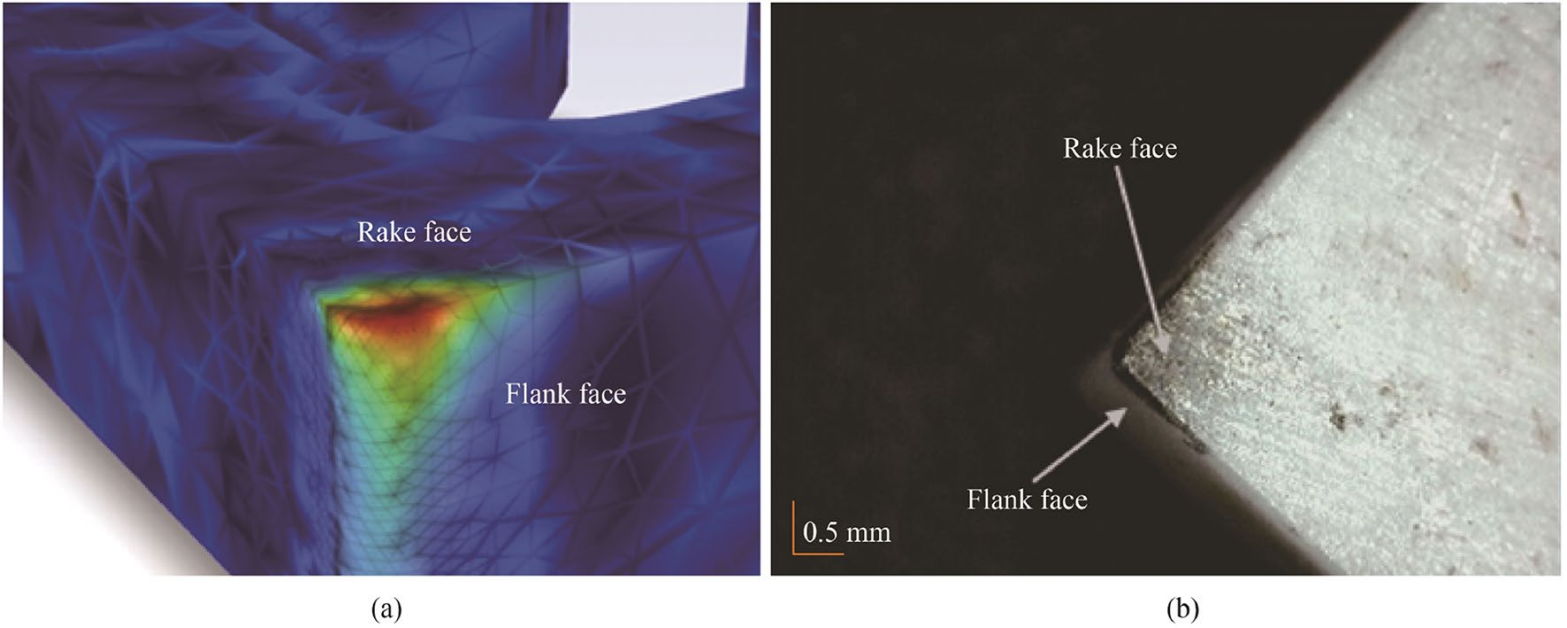
CHT model showing the location of the thermal energy at the region of the cooling channel surface in **a**, **b** corresponding thermal wear pattern on the insert after machining for 10 s at $V_{{\text{c}}}$ = 900 ${\text{m}}/{\text{min}}$

To ensure structural integrity of the tool, the internal channel distance from the flank was limited to a maximum of 0.7 mm, with the distance from the rake face 0.85 mm during machining. The model was constructed to allow for the maximum permissible distance the internal channel could be placed from the tool edge. The liquid gallium responds to thermal changes in the velocity profile in accordance with the local conditions in the cutting zone (see Figs. 30a, b). For example, an increase in cutting speed will invariably increase the local temperature, and in turn, the circulating liquid will experience an input of thermal energy which then produces an increase in the fluidic velocity within the chamber. This has the effect of removing the heat more efficiently through the dynamic convection currents circulating inside the insert.

## Conclusions

This work presented the design and fabrication of an aluminium oxide cutting insert containing an internal channel for enhanced heat removal during machining operations. A design was proposed and analysed using a combinatorial thermomechanical and CFD simulation of the external structure and internal fluidic region. The results indicate that the applied forces and thermal loads are within the structural limitations of the ceramic insert. Using a lithographic ceramic manufacturing method, it was shown that good dimensional reproducibility with complex internal features was possible through additive manufacturing. Controlled sintering produced a densified ceramic body which was subsequently characterised and analysed through a series of tests. Linear fractures were observed in some cases after the application of thermal treatment in initial runs. These were attributed to the presence of structural variations in the preliminary design stage, combined with non-optimised process parameters and densification regimes. The results revealed that there was a strong correlation between the forming parameters and heating regime used in the process. Furthermore, in the case of prototype fabrication, geometry specific modification leading to process optimisation for forming, debinding and sintering, is primarily achieved through trial and error which can be better quantified through further research.

Performance evaluation of the novel internal cooling mechanism indicated internal cooling using liquid gallium reduced localised wear on the tool edge when compared to the same insert under dry machining conditions and also when compared to external cooling with liquid water. The numerical models developed along with the experimental tests results, support the hypothesis that liquid gallium can transfer heat through an internal cooling mechanism in ceramic inserts and in doing so, reduce tool wear.
